# The risk of delirium or dementia‐related hospitalization among individuals living with dementia after long‐term care entry: A population‐based risk prediction model

**DOI:** 10.1002/alz.70487

**Published:** 2025-08-22

**Authors:** Tesfahun C. Eshetie, Gillian E. Caughey, Catherine Lang, Craig Whitehead, Maria Crotty, Megan Corlis, Renuka Visvanathan, Maria C. Inacio

**Affiliations:** ^1^ Registry of Senior Australians Research Centre South Australian Health and Medical Research Institute Adelaide South Australia Australia; ^2^ Registry of Senior Australians Research Centre, Caring Futures Institute, College of Nursing and Health Sciences Flinders University Bedford Park South Australia Australia; ^3^ UniSA Allied Health and Human Performance University of South Australia Adelaide South Australia Australia; ^4^ UniSA Clinical & Health Sciences University of South Australia Adelaide South Australia Australia; ^5^ College of Medicine and Public Health Flinders University Bedford Park South Australia Australia; ^6^ Southern Adelaide Local Health Network SA Health Adelaide South Australia Australia; ^7^ Australian Nursing and Midwifery Federation (SA Branch) Adelaide South Australia Australia; ^8^ Adelaide Geriatrics Training and Research with Aged Care (GTRAC) Centre, Adelaide Medical School, Faculty of Health and Medical Sciences University of Adelaide Adelaide South Australia Australia; ^9^ Aged and Extended Care Services The Queen Elizabeth Hospital and Basil Hetzel Institute for Translational Research, Central Adelaide Local Health Network, SA Health Woodville South South Australia Australia

**Keywords:** delirium, dementia, hospitalization, long‐term care, nursing homes, risk profiling, risk‐prediction

## Abstract

**INTRODUCTION:**

Identifying individuals with dementia in long‐term care facilities (LTCFs) at risk for delirium or dementia‐related hospitalizations can support individualized risk mitigation.

**METHODS:**

Using the Registry of Senior Australians (ROSA) Historical National Cohort (*N* = 207343 individuals with dementia in 2655 LTCFs), we identified predictors of delirium or dementia‐related hospitalization within 365 days of LTCF entry and developed a risk prediction model using elastic net penalized regression and Fine‐Gray model. Model discrimination using area under the receiver operating characteristics curve (AUC), calibration and clinical utility were assessed.

**RESULTS:**

Within 365 days, 5.2% (*N* = 10709) of individuals had a delirium or dementia‐related hospitalization. Forty predictors were identified, strongest included history of frequent emergency department presentations, physical violence history, being male, and prior delirium. Model AUC was 0.664 (95% confidence interval: 0.650–0.676) with reasonable calibration.

**DISCUSSION:**

Our risk prediction model for delirium or dementia‐related hospitalizations had moderate discrimination with reasonable calibration and clinical utility. Routinely collected data can inform risk profiling in LTCFs.

**Highlights:**

- Using a large population‐based cohort of people living with dementia, we developed a risk prediction model for delirium or dementia‐related hospitalization within 365 days of long‐term care facility (LTCF) entry.
- Within 365 days after entry into LTCF, 5.2% of individuals living with dementia had a delirium or dementia‐related hospitalization.
- The model demonstrated moderate discriminatory performance (area under the curve [AUC] = 0.664, 95% confidence interval [CI]: 0.650–0.676) and reasonable calibration in predicting delirium or dementia‐related hospitalization risk.
- Our model showed net benefits within 2%–22% risk threshold ranges assessed via decision curve analysis .
- Risk stratification at LTCF entry may support clinicians and aged care providers in identifying high risk individuals and implementing targeted interventions to reduce delirium or dementia‐related hospitalizations .

## 1 INTRODUCTION

Dementia is a major global public health challenge, with an estimated 57 million people affected worldwide.^1^ As life expectancies increase, this number is expected to triple to 153 million by 2050. In 2021, over 21 million people were estimated to have dementia across the 38 Organization for Economic Co‐operation and Development (OECD) countries,^2^ with more than 421,000 people living with dementia in Australia in 2024.^3^

Cognitive impairment or dementia is common in residents of long‐term care facilities (LTCFs), placing them at a higher risk of hospitalization compared to those without dementia.^4^ LTCF residents living with dementia are also particularly vulnerable to developing delirium or experiencing behavioral changes after LTCF entry.^5^, ^6^ Dementia also significantly reduces the likelihood of recovery following episodes of delirium and is associated with a subsequent declining trajectory of cognitive function.^7^ Hospitalizations due to delirium or dementia among LTCF residents contribute to substantial morbidity, increased mortality, loss of autonomy, a decline in functional status, long‐term cognitive decline, reduced quality of life, and significant health care costs.^8^, ^9^

Reducing unnecessary hospitalizations for people living with dementia is a priority given the medical, social, and economic impacts.^4^ Although many hospitalizations are necessary and can prevent further decline or premature mortality, some may be avoidable with timely and appropriate primary care.^6^ An Australian study found that almost every second hospitalization among people living with dementia was for potentially preventable conditions.^6^ Similarly, in a United States study, potentially preventable hospitalizations were 78% more frequent in people living with dementia, representing 28% of all hospitalizations, compared to just 19% among those without dementia.^5^ Early identification of LTCF residents at high risk of delirium or dementia‐related hospitalizations is one way to reduce the risk of these events.^10^ Delirium, in particular is a potentially modifiable, practice sensitive clinical outcome^11^, ^12^, ^13^ with evidence suggesting that it could be prevented in up to two‐thirds of hospitalized patients through early detection.^14^

While several risk prediction models for delirium have been developed for hospitalized older adults,^15^, ^16^, ^17^, ^18^, ^19^ there are currently no models designed to identify new LTCF residents at greatest risk for delirium or dementia‐related hospitalizations. Few studies have developed models to identify delirium in LCTFs,^20^, ^21^, ^22^ models for behavioral and psychological symptoms of dementia in community‐dwelling individuals,^23^ and tools for stratifying people living with dementia for hospital transfer, but these studies were limited by small sample sizes and did not incorporate factors commonly assessed in LTCF residents, such as neuropsychiatric conditions, cognitive impairment, and mobility needs.

In Australia, extensive information is routinely collected through clinical assessments and administrative data collections when individuals enter long‐term care.^24^ Through data linkage, this information should be efficiently utilized to identify new LTCF residents at risk of poor quality and safety outcomes while in care.^25^, ^26^, ^27^ In this study, using integrated population based aged and health care data, we examined individual, medication, health care, system, and facility‐related factors that are predictors of delirium or dementia‐related hospitalization risk in the 365 days following LTCF entry, and developed a delirium or dementia‐related hospitalization risk prediction model to identify the 365 days risk of these events after LTCF entry.

## 2 METHODS

### 2.1 Study design, setting, and data sources

A retrospective cohort study using the Registry of Senior Australians (ROSA) Historical National Cohort was conducted, which has been previously described.^24^ Briefly, ROSA Historical National Cohort contains information on individuals who have undergone an aged care eligibility assessment and accessed services that require this approval, namely residential care services (either respite or permanent) in LTCFs, home care packages, and transition care.^24^ ROSA integrates national aged care datasets obtained from the Australian Institute of Health and Welfare National Aged Care Data Clearinghouse (NACDC), which includes the National Death Index (NDI), with health care datasets from the Australian Government Medicare Benefits Schedule (MBS), Pharmaceutical Benefits Scheme (PBS), and four states health authorities’ hospitalization data collections (South Australia, New South Wales, Queensland, Victoria). This study utilized specific datasets from the NACDC, namely the aged care eligibility assessment datasets (collected using the “Aged Care Assessment Program” and “National Screening Assessment Form”), the entry into LTCF assessment (collected using the “Aged Care Funding Instrument”), episodes of Residential Aged Care Services, and the NDI. In addition to these datasets, we used admitted and emergency department (ED) hospital data collections from New South Wales, Victoria, Queensland, and South Australia.

**RESEARCH IN CONTEXT**

1. **Systematic review**: While various risk prediction models have been developed for delirium in hospitalized older adults, no models currently exist to identify new long‐term care facility (LTCF) residents at greatest risk for delirium or dementia‐related hospitalizations.
2. **Interpretation**: Using integrated national population‐based aged and health care data, we developed a tailored predictive model for delirium or dementia‐related hospitalization risk within 365 days of LTCF entry, demonstrating moderate discrimination (area under the curve  = 0.664, 95% confidence interval: 0.650–0.676) with reasonable calibration. The decision curve analysis showed that our model has clinical net benefits within risk thresholds of 2%–22%. A history of frequent emergency department presentations, prior behavioral disturbances, history of delirium, use of monoamine oxidase B inhibitors, use of dipeptidyl peptidase 4 inhibitors, complex healthcare needs, and exposure to high sedative load are key predictors of a higher risk of hospitalization for delirium or dementia.
3. **Future directions**: Routinely collected clinical and administrative data during the transition into LTCFs can be leveraged to inform risk profiling for delirium or dementia‐related hospitalizations in individuals living with dementia.

### 2.2 Study cohort

Non‐Indigenous older Australians aged 65–105 years who entered a LTCF in New South Wales, Victoria, Queensland, and South Australia between 01/01/2009 and 31/12/2018 and who were not Department of Veterans’ Affairs concession card holders with a diagnosis of dementia. Dementia was ascertained using health conditions reported in the aged care eligibility or entry into LTCF assessments, or using a history of dispensing of dementia specific medications, including acetylcholinesterase inhibitors or memantine or risperidone for the treatment of behavioral and psychological symptoms of dementia, within the 6 months prior to LTCF entry.^28^ Prior studies suggest that leveraging multiple clinical and administrative data sources improve the accuracy of dementia diagnosis ascertainment.^29^, ^30^

### 2.3 Potential risk predictors

The potential predictors of interest for our risk prediction model included individual, medication, health care, system, and facility‐related factors.

#### 2.3.1 Individual‐level factors

Individual‐level factors were ascertained at the aged care eligibility or entry into LTCF assessments and included: age, sex, preferred language (English vs. other), partner/marital status, Socio‐Economic Indexes for Areas' (SEIFA) relative socio‐economic disadvantage index, SEIFA education and occupation index, SEIFA economic resources index, number of comorbidities (pharmaceutical‐based comorbidity index),^31^ individual health conditions, functional limitations, needs assessment regarding activities of daily living (ADL) (i.e., ratings on nutrition, mobility, personal hygiene, toileting, and continence), behavior (i.e., ratings on cognitive skills, wandering, verbal behavior, physical behavior, and depression), and complex health care needs at entry into care.

#### 2.3.2 Medication‐related factors

Medication‐related factors were ascertained from the pharmaceutical dispensing records 90‐days prior to LTCF entry and included: number of unique medications dispensed, sedative load rating of medications (cumulative effect of medications with sedative properties), specific medication classes at the chemical subgroup World Health Organization Anatomical, Therapeutic and Chemical classification (ATC) fourth level.

#### 2.3.3 Health care‐related factors

Health care‐related factors, ascertained using the history of hospitalizations in the 365 days prior to LTCF entry, included: number of hospitalizations (unplanned and potentially preventable), ED presentations, cumulative length of hospital stays. Additional health care‐related factors were ascertained using the MBS subsidized health encounters in the 365 days prior to LTCF entry and included: primary care (general practitioner [GP] attendances (regular), urgent GP attendances after hours, or GP after hours attendances), specialist or consultant physician attendances (geriatrician, palliative, and pain), health assessments, GP management plans, team care arrangements and multidisciplinary care plans, and comprehensive medications review.

#### 2.3.4 System and facility‐related factors

System and facility‐related factors, ascertained from the LTCF entry assessments and service episodes, included: provider type (not‐for‐profit, for‐profit, or government), facility state, and facility remoteness (major cities, inner regional, outer regional, or remote/very remote).

### 2.4 Outcome of interest

The primary outcome was time to first delirium or dementia‐related hospitalization within 365 days of LTCF entry. Delirium or dementia‐related hospitalizations were identified using hospitalization or ED presentation records where dementia or delirium were the principal discharge diagnoses for the encounter, using International Classification of Diseases, 10th Revision, Australian Modification (ICD‐10‐AM) (Table S1). Evidence indicates that over 50% of residents in Australian LTCFs have dementia,^32^ placing them at increased risk of delirium or dementia‐related hospitalizations, which are potentially preventable. This outcome is one of the 15 quality and safety indicators monitored through the ROSA Outcome Monitoring System (OMS), a national pragmatic quality and safety monitoring and benchmarking system designed to support evidence‐based quality and safety improvement in long‐term care.^33^ These indicators were selected following a comprehensive review of national and international literature and stakeholder consultation, ensuring they are clinically relevant, feasible, technically robust, and capable of driving quality improvement in long‐term care.^33^ All residents were followed for 365 days from LTCF entry to the first delirium or dementia‐related hospitalization, death, exit from LTCF or end of study period (31/12/2019).

### 2.5 Sample size

A convenience sample was used in our study, based on data availability, and no sample size was estimated a priori for our prediction model (Supplementary Methods).

### 2.6 Statistical analysis

Cohort characteristics and outcome measures were summarized using medians, interquartile ranges (IQR), frequencies, and proportions. The study cohort was randomly split into 80% for model training (training sample, *N* = 165,874) and 20% for model testing (testing sample, *N* = 41,469). An elastic net penalized regression approach, using a Fine and Gray model, to determine a regularization path from training datasets was employed.^34^ In this model, we used death as a competing risk.^35^ Analyses were undertaken using the R package fastcmprsk v1.24.5 in RStudio. Elastic net regression was selected for variable selection due to its ability to effectively handle high‐dimensional data with multicollinearity (Supplementary Methods).^36^, ^37^ For further validation and robustness of model development, analyses were conducted independently on the training datasets from each of the four included states (New South Wales, Queensland, Victoria, and South Australia). Predictive factors were selected based on variables retained in at least two state‐specific analyses, with age and sex being exempt from penalization in the model fitting. If a single level of a multi‐level variable was retained in the model, we included all levels (excluding the reference level) to minimize omitted‐variable bias and enhance model interpretability.

Sub‐distribution hazard ratios (sHR) and 95% confidence intervals (CI) were presented. Model discrimination was examined using the area under the receiver operating characteristics curve (AUC) from the 20% testing sample. Model performance was compared to a base model (age and sex only). Model calibration was assessed using calibration plots plotting observed versus deciles of predicted 365 days risk of dementia or delirium‐related hospitalization, for both in‐sample and out‐of‐sample. Decision curve analysis (DCA) was performed to determine the clinical utility of the final and base (based on age and sex) models. We also evaluated the sensitivity, specificity, positive predictive value, and negative predictive value at a variety of clinically relevant risk thresholds to demonstrate the performance of the final model in the testing sample. In a sensitivity analysis, we developed a risk prediction model for delirium‐related hospitalization only (i.e., where delirium was the principal discharge diagnosis for the encounter). We used complete‐case analysis for all calculations due to the small percentage of missing data (< 4.6% of cases). The TRIPOD+AI statement for reporting clinical prediction models were followed (Supplemental TRIPOD+AI Checklist).

### 2.7 Ethics

Approval was obtained from the University of South Australia Human Research Ethics Committee (Ref: 200489), Australian Institute of Health and Welfare Ethics Committee (Ref: EO2022/4/1376), South Australian Department for Health & Wellbeing Human Research Ethics Committee (Ref: HREC/18/SAH/90), and New South Wales Population & Health Services Research Ethics Committee (Ref: 2019/ETH12028).

## 3 RESULTS

### 3.1 Cohort description

**FIGURE 1:**
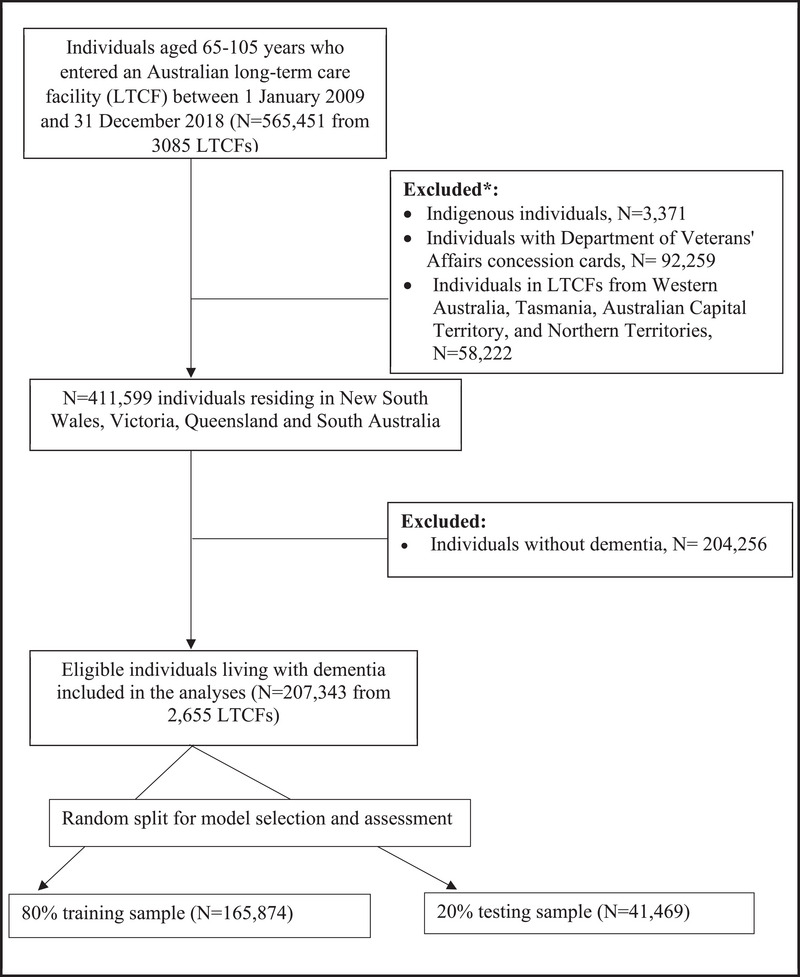
Study cohort flowchart. *Not mutually exclusive.

During the study period, 207,343 individuals living with dementia entered 2,655 LTCFs (Figure 1). The cohort had a median age of 84 years old (IQR 79–88), 124,826 (60.2%) were women, and 72,829 (35.1%) had six or more comorbid conditions. Of the studied cohort, 49,763 (24.0%) residents experienced a high sedative load and 32,095 (15.5%) were on 11 or more unique medications in the 90‐day period prior to entry into LTCFs. Overall, 160,783 (77.6%) had moderate/high ADL needs, 167,812 (80.9%) had moderate/severe behavioral needs, and 47,070 (22.7%) had ≥10 complex health care procedures needed at LTCF entry (Table 1).

**TABLE 1:** Highlights of individual, medication, health care, system, and facility‐related characteristics at entry into LTCFs by delirium or dementia‐related hospitalization.

Characteristics	Total N = 207343 (100.0%)	Delirium or dementia‐related hospitalization within 365 day^a^ N = 10709 (5.2%)	No delirium or dementia‐related hospitalization within 365 days N = 196634 (94.8%)
**Age group, years**			
65–74	22572 (10.9)	1644 (15.4)	20928 (10.6)
75–84	87775 (42.3)	4903 (45.8)	82872 (42.1)
85–94	88784 (42.8)	3915 (36.6)	84869 (43.2)
≥95	8212 (4.0)	247 (2.3)	7965 (4.1)
**Sex**			
Men	82517 (39.8)	5661 (52.9)	76856 (39.1)
Women	124826 (60.2)	5048 (47.1)	119778 (60.9)
**Facility remoteness**			
Major cities	149359 (72.0)	8479 (79.2)	140880 (71.6)
Inner regional	43181 (20.8)	1648 (15.4)	41533 (21.1)
Outer regional	13671 (6.6)	538 (5.0)	13133 (6.7)
Remote or very remote	663 (0.3)	28 (0.3)	635 (0.3)
**SEIFA index of economic resources**			
Q1 (greater disadvantage)	51886 (25.0)	2765 (25.8)	49121 (25.0)
Q2	43531 (21.0)	2038 (19.0)	41493 (21.1)
Q3	44798 (21.6)	2371 (22.1)	42427 (21.6)
Q4	36104 (17.4)	2007 (18.7)	34097 (17.3)
Q5 (lower disadvantage)	28991 (14.0)	1414 (13.2)	27577 (14.0)
**Health conditions** ^b^			
Incontinence (urinary or bowel)	85699 (41.3)	4383 (40.9)	81316 (41.4)
History of falls	68885 (33.2)	3407 (31.8)	65478 (33.3)
Abnormalities of gait & mobility	60805 (29.3)	2693 (25.1)	58112 (29.6)
Osteoporosis	46969 (22.7)	1988 (18.6)	44981 (22.9)
History of fractures	45934 (22.2)	1911 (17.8)	44023 (22.39)
Type 2 diabetes mellitus	43170 (20.8)	2578 (24.1)	40592 (20.6)
Psychoses	42653 (20.6)	2953 (27.6)	39700 (20.2)
Disorientation	30941 (14.9)	1837 (17.2)	29104 (14.8)
Acute and chronic ischaemic heart disease	29932 (14.4)	1743 (16.3)	28189 (14.3)
Parkinson's disease	15568 (7.5)	902 (8.4)	14666 (7.5)
History of delirium	15420 (7.4)	1228 (11.5)	14192 (7.2)
Breathing difficulties/shortness of breath	8283 (4.0)	352 (3.3)	7931 (4.0)
Restlessness & agitation	9475 (4.6)	808 (7.5)	8667 (4.4)
Other mental & behavioral disorders	4793 (2.3)	376 (3.5)	4417 (2.2)
Epilepsy	4594 (2.2)	286 (2.7)	4308 (2.2)
Irritability & anger	4121 (2.0)	333 (3.1)	3788 (1.9)
Mental and behavioral disorders due to alcohol and other psychoactive substance use	3454 (1.7)	172 (1.6)	3282 (1.7)
History of physical violence	701 (0.3)	101 (0.9)	600 (0.3)
**Rx‐Risk‐V co‐morbidity category**			
0–1	26992 (13.0)	1253 (11.7)	25739 (13.1)
2–3	48323 (23.3)	2300 (21.5)	46023 (23.4)
4–5	59199 (28.6)	2955 (27.6)	56244 (28.6)
6–8	56924 (27.5)	3209 (30.0)	53715 (27.3)
≥9	15905 (7.7)	992 (9.3)	14913 (7.6)
**Sedative loading rating**			
0	76841 (37.1)	3315 (31.0)	73526 (37.4)
1–2	80739 (38.9)	4131 (38.6)	76608 (39.0)
≥3	49763 (24.0)	3263 (30.5)	46500 (23.6)
**No. of unique medications**			
0‐4	76940 (37.1)	3773 (35.2)	73167 (37.2)
5‐10	98308 (47.4)	5098 (47.6)	93210 (47.4)
11+	32095 (15.5)	1838 (17.2)	30257 (15.4)
**Specific medications** ^c^			
Benzodiazepine derivatives (N05BA)	20369 (9.8)	1491 (13.9)	18878 (9.6)
Diazepines, oxazepines, thiazepines, and oxepines (N05AH)	13538 (6.5)	1091 (10.2)	12447 (6.3)
Propulsives (prokinetic gastrointestinal motility medications) (A03FA)	9892 (4.8)	405 (3.8)	9487 (4.8)
Direct factor Xa inhibitors (B01AF)	8206 (4.0)	530 (4.9)	7676 (3.9)
Fatty acid derivative antiepileptics (N03AG)	6513 (3.1)	518 (4.8)	5995 (3.0)
Dipeptidyl peptidase 4 (DPP‐4) inhibitors (A10BH)	3206 (1.5)	233(2.2)	2973 (1.5)
Other antiepileptics (N03AX)	3159 (1.5)	231 (2.2)	2928 (1.5)
Monoamine oxidase type B inhibitors (N04BD)	832 (0.4)	74 (0.7)	758 (0.4)
**ADL level** ^e^			
No or minimal impairment	2945 (1.4)	103 (1.0)	2842 (1.4)
Mild impairment	42403 (20.5)	1911 (17.8)	40492 (20.6)
Moderate impairment	69571 (33.6)	3902 (36.4)	65669 (33.4)
High impairment	91212 (44.0)	4740 (44.3)	86472 (44.0)
**Behavioral daily living level** ^e^			
No or minimal impairment	6963 (3.4)	180 (1.7)	6783 (3.4)
Mild impairment	31356 (15.1)	1048 (9.8)	30308 (15.4)
Moderate impairment	44173 (21.3)	1731 (16.2)	42442 (21.6)
High impairment	123639 (59.6)	7697 (71.9)	115942 (59.0)
**Complex health care rating** ^d^, ^e^			
A (best)	49187 (23.7)	2708 (25.3)	46479 (23.6)
B	51288 (24.7)	2498 (23.3)	48790 (24.8)
C	58586 (28.3)	3115 (29.1)	55471 (28.2)
D (worst)	47070 (22.7)	2335 (21.8)	44735 (22.8)
**Mobility rating** ^d^, ^e^			
A (best)	9767 (4.7)	459 (4.3)	9308 (4.7)
B	15772 (7.6)	757 (7.1)	15015 (7.6)
C	85329 (41.2)	4835 (45.1)	80494 (40.9)
D (worst)	95263 (45.9)	4605 (43.0)	90658 (46.1)
**Wandering rating** ^d^, ^e^			
A (best)	131123 (63.2)	5833 (54.5)	125290 (63.7)
B	19890 (9.6)	980 (9.2)	18910 (9.6)
C	13103 (6.3)	725 (6.8)	12378 (6.3)
D (worst)	42015 (20.3)	3118 (29.1)	38897 (19.8)
**Verbal behavior rating** ^d^, ^e^			
A (best)	34321 (16.6)	1215 (11.3)	33106 (16.8)
B	31881 (15.4)	1481 (13.8)	30400 (15.5)
C	34481 (16.6)	1733 (16.2)	32748 (16.7)
D (worst)	105448 (50.9)	6227 (58.1)	99221 (50.5)
**Physical behavior rating** ^d^, ^e^			
A (best)	56485 (27.2)	2022 (18.9)	54463 (27.7)
B	32455 (15.7)	1641 (15.3)	30814 (15.7)
C	30005 (14.5)	1530 (14.3)	28475 (14.5)
D (worst)	87186 (42.0)	5463 (51.0)	81723 (41.6)
**No. of unplanned hospitalizations** ^f^			
None	64002 (30.9)	2847 (26.6)	61155 (31.1)
1	69755 (33.6)	3506 (32.7)	66249 (33.7)
2‐4	64616 (31.2)	3701 (34.6)	60915 (31.0)
≥5	8970 (4.3)	655 (6.1)	8315 (4.2)
**No. of ED presentations** ^f^			
0	67254 (32.4)	2745 (25.6)	64509 (32.8)
1	60812 (29.3)	3097 (28.9)	57715 (29.4)
2‐4	65395 (31.5)	3837 (35.8)	61558 (31.3)
≥5	13882 (6.7)	1030 (9.6)	12852 (6.5)
**No. of GP attendances** ^f^			
0	7049 (3.4)	323 (3.0)	6726 (3.4)
1–5	54819 (26.4)	2491 (23.3)	52328 (26.6)
6–15	99616 (48.0)	5277 (49.3)	94339 (48.0)
≥16	45859 (22.1)	2618 (24.4)	43241 (22.0)
**No. of geriatric medicine physician attendances** ^f^			
0	184433 (89.0)	9332 (87.1)	175101 (89.0)
1	15136 (7.3)	906 (8.5)	14230 (7.2)
≥2	7774 (3.7)	471 (4.4)	7303 (3.7)

Missing data *N*(%): Facility Remoteness: 469(0.2); SEIFA Index of Economic Resources: 2033(1.0).

Abbreviations: ADL, activities of daily living; ED, emergency department; GP, general practitioner; LTCFs, long‐term care facilities.

^a^ Delirium or dementia‐related hospitalisation within 365 days after LTCF entry, defined as hospitalisation or ED presentation where dementia or delirium was recorded as the principal discharge diagnosis for the encounter.

^b^ Conditions were ascertained using the aged care eligibility or entry into LTCF assessments.

^c^ Medications at the chemical subgroup level (ATC 4th level, i.e., 5 digits).

^d^ A is least dependent and D is most dependent.

^e^ Care needs of LTCF residents from entry into LTCF assessment : missing data N(%): 1212(0.6).

^f^ Ascertained in the 365 days prior to LTCF entry.

Within 365 days after entry into LTCF, 10,709 (5.2%) individuals living with dementia had a delirium or dementia‐related hospitalization, and 6612 (3.2%) individuals experienced a delirium‐related hospitalization only (Table 1). The cumulative incidence of death as a competing risk within 365 days was 25.5% (95% CI: 25.3%–25.7%).

Compared to individuals living with dementia who did not have a delirium or dementia‐related hospitalization after LTCF entry, those who had a hospitalization had a higher prevalence of psychoses (27.6% vs. 20.2%), history of delirium (11.5% vs. 7.2%), type 2 diabetes mellitus (24.1% vs. 20.6%), exposure to high sedative load (30.5% vs. 23.6%), and high behavioral daily living care needs (71.9% vs. 59.0%) (Table 1). Tables S2–S6 have detailed individual, medication, health care, system, and facility‐related characteristics not in Table 1. Tables S7 and S8 provide detailed characteristics for the training and testing samples employed for model development and testing.

### 3.2 Predictors of delirium or dementia‐related hospitalization risk within 365 days of LTCF entry

For people living with dementia, a comprehensive set of 40 variables contributed to delirium or dementia‐related hospitalization risk within 365 days of LTCF entry (Table 2).

**TABLE 2:** Predictors of delirium or dementia‐related hospitalization within 365 days of entry into LTCFs.

Variables	sHR	95% CI	p‐value
Age at LTCF entry	0.98	0.98–0.99	<0.001
Men vs. women	1.57	1.49–1.65	<0.001
Born in Australia	0.94	0.89–0.99	0.028
Preferred language (English vs. other)	0.90	0.84–0.97	0.007
Facility remoteness			
Inner regional vs. major cities	0.71	0.66–0.76	<0.001
Outer regional vs. major cities	0.78	0.70–0.86	<0.001
Remote or very remote vs. major cities	0.86	0.56–1.34	0.510
Provider type			
Government vs. not‐for‐profit	0.69	0.60–0.80	<0.001
Private vs. not‐for‐profit	1.06	1.02–1.11	0.004
SEIFA Index of Economic Resources Quintile			
Q2 vs. Q1	0.93	0.87–0.99	0.034
Q3 vs. Q1	0.96	0.90–1.03	0.230
Q4 vs. Q1	0.99	0.92–1.06	0.770
Q5 vs. Q1	0.89	0.83–0.96	0.002
History of delirium (yes vs. no)	1.48	1.38–1.60	<0.001
Restlessness and agitation (yes vs. no)	1.21	1.12–1.31	<0.001
History of physical violence (yes vs. no)	1.64	1.30–2.06	<0.001
Irritability and anger (yes vs. no)	1.27	1.13–1.43	<0.001
Breathing difficulties/shortness of breath (yes vs. no)	0.89	0.79–1.00	0.058
Disorientation (yes vs. no)	1.12	1.06–1.18	<0.001
Abnormalities of gait and mobility (yes vs. no)	0.87	0.83–0.91	<0.001
Other mental & behavioral disorders (yes vs. no)^a^	1.30	1.14–1.47	<0.001
Mental and behavioral disorders due to alcohol and other psychoactive substance use (yes vs. no)	0.68	0.55–0.84	<0.001
Acute and chronic ischemic heart disease (yes vs. no)	1.09	1.03–1.16	0.004
Psychoses (yes vs. no)	1.08	1.02–1.15	0.011
Benzodiazepine derivatives (N05BA^b^) (yes vs. no)	1.23	1.13–1.33	<0.001
Diazepines, oxazepines, thiazepines, and oxepines (as antipsychotics) (N05AH^b^) (yes vs. no)	1.09	1.01–1.19	0.036
Propulsives (A03FA^b^) (yes vs. no)	0.79	0.70–0.88	<0.001
Monoamine oxidase type B inhibitors (N04BD^b^) (yes vs. no)	1.43	1.07–1.90	0.014
Dipeptidyl peptidase 4 (DPP‐4) inhibitors (A10BH^b^) (yes vs. no)	1.34	1.15–1.55	<0.001
Fatty acid derivative antiepileptics (N03AG^b^) (yes vs. no)	1.15	1.03–1.28	0.015
Direct factor Xa inhibitors (B01AF^b^) (yes vs. no)	1.19	1.07–1.32	0.001
Other antiepileptics (N03AX^b^) (yes vs. no)	1.25	1.09–1.42	<0.001
Sedative loading rating			
1‐2 vs. 0	1.05	1.00–1.11	0.057
≥3 vs. 0	1.16	1.07–1.24	<0.001
Complex health care needs level^c^			
No or minimal vs. severe impairment	0.70	0.60–0.82	<0.001
Mild vs. severe impairment	0.89	0.78–1.01	0.060
Moderate vs. severe impairment	0.99	0.90–1.09	0.880
Behavioral daily living level			
No or minimal vs. severe impairment	0.96	0.76–1.23	0.760
Mild vs. severe impairment	0.94	0.84–1.06	0.290
Moderate vs. severe impairment	0.88	0.81–0.96	0.004
Complex health care rating^d^			
A best vs. D worst	1.40	1.22–1.60	<0.001
B vs. D worst	1.16	1.02–1.32	0.026
C vs. D worst	1.05	0.96–1.15	0.320
Mobility rating^d^			
A best vs. D worst	1.15	1.01–1.31	0.037
B vs. D worst	1.20	1.09–1.32	<0.001
C vs. D worst	1.17	1.11–1.24	<0.001
Wandering rating^d^			
A best vs. D worst	0.79	0.74–0.85	<0.001
B vs. D worst	0.88	0.81–0.97	0.008
C vs. D worst	0.89	0.80–0.98	0.014
Verbal behavior rating^d^			
A best vs. D worst	0.81	0.74–0.89	<0.001
B vs. D worst	0.94	0.87–1.01	0.076
C vs. D worst	0.94	0.88–1.01	0.095
Physical behavior rating^d^			
A best vs. D worst	0.83	0.76–0.90	<0.001
B vs. D worst	1.03	0.94–1.12	0.540
C vs. D worst	0.93	0.87–0.99	0.031
Cognitive rating^d^			
A best vs. D worst	0.67	0.57–0.78	<0.001
B vs. D worst	0.64	0.59–0.69	<0.001
C vs. D worst	0.82	0.78–0.86	<0.001
No. of unplanned hospitalizations^e^			
1 vs. 0	0.95	0.88–1.03	0.240
2‐4 vs. 0	1.00	0.91–1.09	0.940
≥5 vs. 0	1.12	0.98–1.27	0.110
No. of ED presentations^e^			
1 vs. 0	1.27	1.18–1.37	<0.001
2‐4 vs. 0	1.41	1.29–1.53	<0.001
≥5 vs. 0	1.65	1.47–1.84	<0.001
No. of GP attendances^e^			
1‐5 vs. 0	1.03	0.88–1.19	0.720
6‐15 vs. 0	1.18	1.02–1.36	0.024
≥16 vs. 0	1.24	1.06–1.46	0.007
No. of geriatric medicine attendances^e^			
1 vs. 0	1.10	1.02–1.19	0.009
≥2 vs. 0	1.10	1.02–1.20	0.019
No. of GP management plans, team care arrangements, multidisciplinary care plans^e^			
1 vs. 0	0.91	0.84–0.98	0.019
≥2 vs. 0	1.06	1.01–1.11	0.019

Abbreviations: CI, confidence intervals; ED, emergency department; GP, general practitioner; LTCF, long‐term care facilities; SEIFA, socio‐economic indexes for areas; sHR, subdistribution hazard ratio.

^a^ Other mental and behavioral disorders include equivalent International Classification of Diseases, Tenth Revision, Australian Modification (ICD‐10‐AM) codes: F10.0–F10.6, F10.8–19, F60–69, F98.5, F07, F50–52, F54–55, F59, and F99.

^b^ Medications at the chemical subgroup level (ATC 4th level, i.e., 5 digits), ascertained from the PBS dispensing records in the 90 days prior to entry into LTCF.

^c^ Complex Health Care Needs level describe the combination of ratings for medication assistance and complex health care procedures (i.e., A, B, C, or D rating applied to a matrix equals the score for the complex care level: Low = 1, Medium = 2, and High = 3).

^d^ A is least dependent and D is most dependent.

^e^ Ascertained in the 365 days prior to LTCF entry.

#### 3.2.1 Individual‐level predictors

Individual‐level predictors of delirium or dementia‐related hospitalization risk within 365 days of LTCF entry were: history of physical violence (yes vs. no, sHR = 1.64, 95% CI: 1.30–2.06), being a man (sHR = 1.57, 95% CI: 1.49–1.65), history of delirium (yes vs. no, sHR = 1.48, 95% CI: 1.38–1.60), complex health care rating (none vs. high care needs, sHR = 1.40, 95% CI: 1.22–1.60), other mental and behavioral disorders (yes vs. no, sHR = 1.30, 95% CI: 1.14–1.47), irritability and anger (yes vs. no, sHR = 1.27, 95% CI: 1.13–1.43), restlessness and agitation (yes vs. no, sHR = 1.21, 95% CI: 1.12–1.31), mobility rating (none or minimal vs. high care needs, sHR = 1.15, 95% CI: 1.01–1.31; low vs. high care needs, sHR = 1.20, 95% CI: 1.09–1.32; moderate vs. high care needs, sHR = 1.17, 95% CI: 1.11–1.24) (Table 2).

Preferred language (English vs. other, sHR = 0.90, 95% CI: 0.84–0.97), being born in Australia (sHR = 0.94, 95% CI: 0.89–0.99), SEIFA Index of Economic Resources (Q1[lower] vs. Q5 [greater], sHR = 0.89, 95% CI: 0.83–0.96), abnormalities of gait and mobility (yes vs. no, sHR = 0.87, 95% CI: 0.83–0.91), mental and behavioral disorders due to alcohol and other psychoactive substance use (yes vs. no, sHR = 0.68, 95% CI: 0.55–0.84), and age at LTCF entry (one unit increase, sHR = 0.98, 95% CI: 0.98–0.99) were associated with a lower risk of delirium or dementia‐related hospitalization. By comparison to individuals with high care needs for cognition, verbal behavior, physical behavior, or wandering, those with no or minimal care needs (cognitive skills, sHR = 0.67, 95% CI: 0.57–0.78; verbal behavior, sHR = 0.81, 95% CI: 0.74–0.89); physical behavior, sHR = 0.83, 95% CI: 0.76–0.90); wandering, sHR = 0.79, 95% CI: 0.74–0.85), low (cognitive skills, sHR = 0.64, 95% CI: 0.57–0.78; wandering, sHR = 0.88, 95% CI: 0.81–0.97), medium (cognitive skills, sHR = 0.82, 95% CI: 0.78–0.86; wandering, sHR = 0.89, 95% CI: 0.80–0.98) were less likely to have a delirium or dementia‐related hospitalization. Compared to individuals with higher care needs, those with no or minimal (sHR = 0.70, 95% CI: 0.60–0.82), moderate (sHR = 0.88, 95% CI: 0.81–0.96) care needs for complex health care and behavioral daily living level, respectively, were less likely to have a delirium or dementia‐related hospitalization (Table 2).

#### 3.2.2 Medication‐related predictors

Medication‐related predictors of delirium or dementia‐related hospitalization risk within 365 days of LTCF entry included: use of monoamine oxidase B inhibitor medications (sHR = 1.43, 95% CI: 1.07–1.90), use of dipeptidyl peptidase 4 (DPP‐4) inhibitor medications (sHR = 1.34, 95% CI 1.15–1.55), use of other antiepileptic medications (e.g., gabapentin, sHR = 1.25, 95% CI: 1.09–1.42), use of direct factor Xa inhibitor medications (sHR = 1.19, 95% CI: 1.07–1.32), sedative load rating (high vs. none, sHR = 1.16, 95% CI: 1.07–1.24), use of fatty acid derivative antiepileptic medications (e.g., valproate, sHR = 1.15, 95% CI: 1.03–1.28), and use of propulsive medicines (prokinetic gastrointestinal motility medicines, e.g., metoclopramide) (sHR = 0.79, 95% CI: 0.70–0.88) (Table 2).

#### 3.2.3 Health care‐related predictors

History of ED presentations (greater than or equal to five attendances vs. none, sHR = 1.65, 95% CI: 1.47–1.84; two to four attendances vs. none, sHR = 1.41, 95% CI: 1.29–1.53; one attendance vs. none, sHR = 1.27, 95% CI: 1.18–1.37), number of GP attendances (≥16 attendances vs. none, sHR = 1.24, 95% CI 1.06–1.46; 6–15 attendances vs. none, sHR = 1.18, 95% CI: 1.02–1.36), and number of geriatric medicine attendances (≥2 attendances vs. none, sHR = 1.10, 95% CI: 1.02–1.20; one vs. none, sHR = 1.10, 95% CI: 1.02–1.19) were health care‐related predictors of delirium or dementia‐related hospitalization (Table 2).

#### 3.2.4 System‐ and facility‐related predictors

These predictors included LTCF ownership type (government vs. not‐for‐profit, sHR = 0.69, 95% CI: 0.60–0.80) and facility remoteness (inner regional vs. major cities, sHR = 0.71, 95% CI: 0.66–0.76; outer regional vs. major cities, sHR = 0.78, 95% CI: 0.70–0.86).

### 3.3 Delirium or dementia‐related hospitalization risk prediction model performance

**FIGURE 2:**
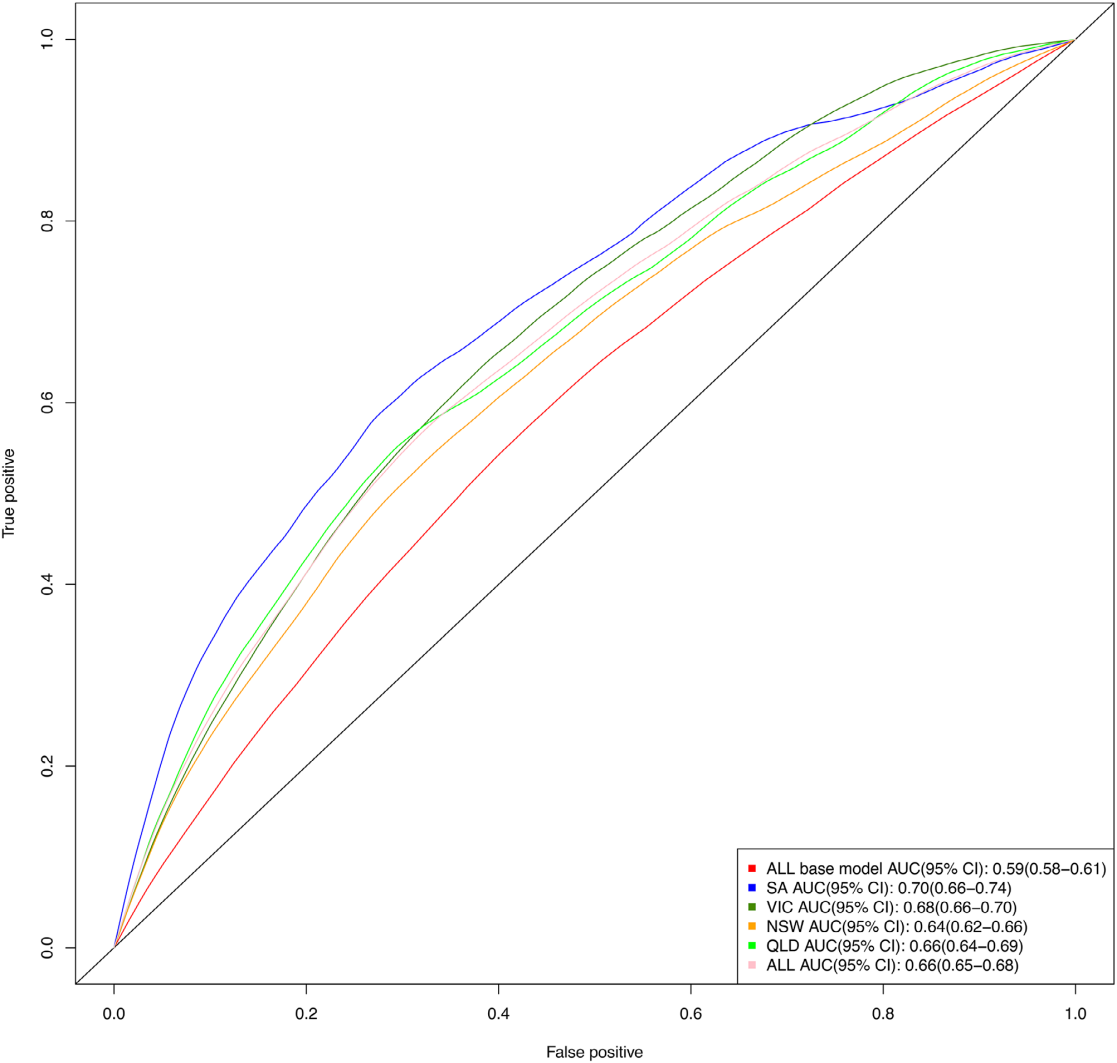
The predictive performance of the delirium or dementia‐related hospitalization risk prediction model (testing sample). AUC, area under the receiver operating characteristics curve; CI, confidence interval; NSW, New South Wales; QLD, Queensland; SA, South Australia; VIC, Victoria.

The discriminative power (AUC) of the risk prediction model in the testing cohort (*N* = 41,469) was  0.664 (95% CI: 0.650–0.676) (Training vs. testing model performance, Supporting Information). This was an improvement from a base (age and sex) prediction model (AUC = 0.592, 95% CI: 0.581–0.606) (Figure 2). Model calibration was assessed both in the training (Figure S1) and testing sample (Figure S2) using calibration plots based on deciles of predicted risk, indicating that the model was reasonably calibrated, with an overestimation in the upper deciles of predicted risk in the testing sample. The final model showed clinical utility with a net benefit within risk thresholds of 2%–22%, which is better than the base model (net benefits within 3%–12.5% risk thresholds) (Figure S3). The sensitivity, specificity, positive predictive value, and negative predictive value across a range of clinically relevant risk thresholds are presented in Table S9. At 5% threshold, which was selected as optimal balancing sensitivity and specificity, the model achieved a sensitivity of 73.6%, specificity of 46.7%, positive predictive value of 6.9%, and negative predictive value of 97.0%. In the sensitivity analysis, the risk prediction model for delirium‐related hospitalization only demonstrated similar performance to the primary model (AUC  = 0.63,95% CI: 0.62–0.65, Figure S4).

## 4 DISCUSSION

Using a large population‐based sample of people living with dementia, we developed a risk prediction model for delirium or dementia‐related hospitalization within 365 days of LTCF entry. The model demonstrated a moderate discriminative ability with significant improvement over the base model that included only age and sex. Our model showed clinical net benefits within risk thresholds of 2%–22% in the DCA, demonstrating clinical value of the model. Our study identified 40 factors contributing to delirium or dementia‐related hospitalization risk within 365 days following entry to LTCF in residents living with dementia. In particular, factors that can be used to identify those at increased risk of hospitalization for delirium or dementia included a prior history of behavioral disturbances (such as aggression, irritability, anger, restlessness and agitation), use of monoamine oxidase B inhibitors, use of DPP‐4 inhibitors, exposure to a high sedative load, use of benzodiazepines, use of antipsychotics, frequent GP and geriatric medicine attendances.

### 4.1 Predictors of delirium or dementia‐related hospitalization risk within 365 days of LTCF entry

Among new LTCF residents living with dementia 5.2% had at least one hospitalization for delirium or dementia within 365 days of care entry. Our estimate is consistent with a prior study using Australia's two most populous states and the ROSA data, which reported the annual incidence of delirium‐related hospitalization between 4.7% (95% CI: 4.6–4.9) in 2011–2012 and 6.7% (95% CI: 6.5–6.8) in 2015–2016.^38^ In our study, 3.2% of individuals experienced a delirium‐related hospitalization only, which is comparable to the 3.1% delirium incidence estimates reported in a recent Canadian population‐based study which ascertained the events via a delirium clinical assessment protocol at entry into care.^7^

In our study, several established predictors for delirium or dementia‐related hospitalization risk were confirmed, including male sex, language, history of delirium, prior history of ED presentations, behavioral disturbances, exposure to high sedative load, use of benzodiazepines, and use of antipsychotics (i.e., clozapine, olanzapine, and quetiapine).^4^, ^6^, ^38^, ^39^ Among these, prior history of frequent ED presentations, history of physical violence, male sex, and prior history of delirium were the strongest predictors identified. In Australia, the Australian Institute of Health and Welfare’s report shows that men had a higher age‐standardized rate of hospitalizations than women (49.7 vs. 36.2 per 10,000 population 60 years and over).^40^ History of physical violence, care needs for wandering, verbal or physical behavior, and use of psychotropic medicines (i.e., benzodiazepines, antipyschotics, and antidepressant) also contributed to risk of delirium or dementia‐related hospitalization. This is consistent with previous studies that reported behavioral and psychological symptoms of dementia dominates the clinical presentation of LTCF residents living with dementia, with 29%–90% of residents in Australian LTCFs experiencing behavioral and psychological symptoms of dementia.^40^ Our findings support how care needs assessment at time of LTCF entry are important in identifying LTCF residents at high risk of hospitalization for delirium or dementia and therefore should be leveraged to better target and personalize interventions. Similarly, we found that increasing numbers of ED presentations in the 365 days prior to entering LTCFs was associated with higher risk of hospitalization for delirium or dementia among new LTCF residents, consistent with previous evidence.^38^ Individuals with frequent ED presentations could be flagged for comprehensive health assessments by GPs,^41^ outreach/in‐reach multidisciplinary services^42^, ^43^, ^44^ to address underlying issues contributing to frequent ED presentation. In addition to ED presentations, certain health care utilization‐related factors were predictors of delirium or dementia‐related hospitalization. Specifically, frequent geriatric medicine attendance was associated with increased hospitalization risk, likely due to improved detection of unmet health care needs prompting an increase in hospitalization. Conversely, LTCF residents with 1‐year prior history of GP management plans, team care arrangements, and multidisciplinary care plans had variable impact on delirium or dementia‐related hospitalization risk, with one episode versus none associated with a lower hospitalization risks whereas two or more episodes of care plan were associated with a higher risk of hospitalization, likely due to improved detection of events such as delirium prompting an increase in hospitalization. This observation is consistent with prior literature about management plans in people with chronic diseases, including in individuals with stroke.^45^

While several delirium risk prediction models exist for hospitalized older adults, few considered medication‐related factors in their development.^46^ In our study, several classes of medicines were analyzed at the chemical subgroup level (ATC fourth level), and exposure to high sedative load, use of benzodiazepines, monoamine oxidase type B inhibitors, antipsychotics (i.e., quetiapine), antiepileptic medicines (i.e., gabapentin, valproate), direct factor Xa inhibitor anticoagulant, and DPP‐4 inhibitors were identified as predictors of hospitalization for delirium or dementia. It is not unexpected that exposure to a high sedative load, psychotropic medicines, and other medicines with sedative properties are associated with these events, likely because of their documented increased risk of delirium. Comprehensive medication reviews targeting these high‐risk medicines (i.e., benzodiazepines, antipsychotics, antidepressants, and anticoagulants) may offer benefit in reducing these events. DPP‐4 inhibitors specifically, are likely linked to hypoglycaemic episodes, which is a potential trigger for delirium.^47^

Being born in Australia, having lower cognitive and behavioral care needs, and entering a government owned LTCF were identified as factors associated with a lower risk of hospitalization. These findings highlight the need for better understanding and support of our population of culturally and linguistically diverse backgrounds and confirms findings of another recent Australian study that identified language was a significant predictor of potentially avoidable admissions due to ambulatory care sensitive conditions for people living with dementia.^6^ Our findings also highlight that the assessments undertaken at entry into LTCFs are robust in identifying those with higher care needs in this area, which can and should be used for care planning to implement tailored interventions to meet individual needs effectively. Finally, facility‐level factors play a critical role in determining delirium or dementia‐related hospitalization risk, and this agrees with previous national evidence that government‐owned LTCFs delivered higher care quality in a number of areas.^48^, ^49^, ^50^

### 4.2 Delirium or dementia‐related hospitalization risk assessment model performance

Our risk prediction model had moderate discriminatory ability with reasonable calibration. Given our objective of identifying individuals at high risk of dementia or delirium‐related hospitalization to support targeted preventive interventions, a predicted risk threshold of 5% was identified as optimal cut‐off (sensitivity: 73.6%, specificity: 46.7%, negative predictive value: 97.0%, and positive predictive value: 6.9%). This threshold offers a reasonable trade‐off between sensitivity and specificity, capturing a substantial proportion of true high‐risk cases (i.e., captures nearly three of four high‐risk individuals) while limiting the burden of false positives. However, the choice of the threshold cut‐off may justifiably differ across LTC settings based on local priorities, resource availability, and risk tolerance. While we did not identify prior published models estimating delirium or dementia‐related hospitalization risk after LTCF entry, our model's discriminative ability is comparable to a model for delirium prediction developed in LTCFs in Spain (AUC = 0.72, 95% CI: 0.66–0.78)^22^ and is lower than a 30‐day risk prediction model for potentially preventable hospitalization among community‐dwelling older adults living with dementia in the United States (AUC = 0.83, 95% CI:  0.82–0.84).^51^ The improvement in our model performance when adding a number of variables beyond age and sex highlights the multifactorial nature of hospitalization risk in this vulnerable population. Given that delirium or dementia‐related hospitalization is a practice‐sensitive quality of care indicator^11^, ^33^ and over 70,000 individuals enter LTCFs each year, of which about half have dementia,^32^ our model presents an opportunity to identify at‐risk individuals during this critical transition period. With limited evidence on interventions for preventing delirium in LTCFs,^14^ our model could be a potential tool in assisting health and aged care providers. Our long‐term goals include the development of a user‐friendly web‐based application tool for risk profiling that can be accessed by aged care and health care professionals for all individuals entering long‐term care.

### 4.3 Limitations

Our study has some limitations. First, although our study included a nationally representative cohort of new LTCF residents from four Australian states (∼87% of the Australian population in LTCFs), hospitalizations in private hospitals for the state of South Australia (43.4% of the cases) were not available and there maybe under‐reporting of delirium or dementia‐related hospitalizations. However, public hospitals capture an estimated 92% of emergency admissions in Australia, and about 4% of all non‐admitted events occur in private hospitals.^52^ Second, it is possible that differences in coding practices across states hospitalization data collections may have affected our identification of the events of interest. Third, other potential predictors of the events of interest, including facility‐related factors, such as staffing levels and other workforce‐related factors (e.g. nursing hours, continuity of primary care),^11^ which have been identified as potential contributors to the events studied, were not able to be included in our analyses due to lack of availability. Future work should explore ways to improve discrimination (e.g., incorporating these potential workforce‐related factors when available) and assess clinical utility using DCA across diverse long‐term care settings. Other limitations include the exclusion of Department of Veterans’ Affairs concession card holders, who access MBS‐funded services differently, and the focus on non‐indigenous older Australians due to our ethics approvals. Additionally, given that our goal of this study was to develop a risk prediction model, the relationship between predictors included in our model and the outcome do not allow for causal inferences.

## 5 CONCLUSION

Our study identified a comprehensive set of 40 factors contributing to delirium or dementia‐related hospitalization risk, prior history of frequent ED presentations, history of physical violence, male sex, and prior history of delirium being the strongest predictors of delirium or dementia‐related hospitalization. Our study developed a tailored predictive model for delirium or dementia‐related hospitalization within 365 days of LTCF entry using national population‐based data collections. The model's moderate discriminative power with reasonable calibration and net benefits in a decision curve analysis suggest it could be a valuable tool for identifying high‐risk individuals who may benefit from targeted preventive strategies. Our model could support health and aged care providers to reduce the burden of delirium or dementia‐related hospitalization, which in turn could positively impact quality of life, morbidity, and mortality for people living with dementia.

## CONFLICT OF INTEREST STATEMENT

The authors declare no conflicts of interest. Author disclosures are available in the Supporting Information.

## CONSENT STATEMENT

The ethics approvals listed for this study were granted on the basis of a waiver of consent pursuant to s.95 of the Australian Privacy Act 1988 (Cth) and other state specific legislation.

## Supporting information



Supporting information

Supporting information
