# Supplementary material for: The risk of delirium or dementia‐related hospitalization among individuals living with dementia after long‐term care entry: A population‐based risk prediction model
Source: Alzheimers Dement. 2025 Aug 22;21(8):e70487. doi: 10.1002/alz.70487 (PMC12371554; doi:10.1002/alz.70487)
Supplement: Supplementary file 2 — Supporting information [file ALZ-21-e70487-s002.docx]

**Supplementary**

**Table of Contents**

[Supplementary Methods 2](#_Toc200633743)

[Supplemental Table 1. International Classification of Diseases, 10th Revision, Australian Modification (ICD-10-AM) Codes to Identify Delirium or Dementia-related Hospitalisation. 3](#_Toc200633744)

[Supplemental Table 2. Individual Characteristics at Entry into Long Term Care Facilities by Delirium or Dementia-related Hospitalisation Status Within 365 Days of Entry (Not Included in Table 1). 4](#_Toc200633745)

[Supplemental Table 3. Individual and Facility-related Characteristics at Entry into Long Term Care Facilities from Aged Care Funding Instrument Assessment^*^ by Delirium or Dementia-related Hospitalisation Status Within 365 Days of Entry (Not Included in Table 1). 7](#_Toc200633746)

[Supplemental Table 4. Medication-related *C*haracteristics in the 90 Days Prior to Entry into Long Term Care Facilities by Delirium or Dementia-related Hospitalisation Status Within 365 Days of Entry (Not Included in Table 1). 9](#_Toc200633747)

[Supplemental Table 5. Hospital-related Health Care Utilisation Within One Year Prior of Entry into Long Term Care Facilities by Delirium or Dementia-related Hospitalisation Within 365 Days of Entry (Not Included in Table 1). 11](#_Toc200633748)

[Supplemental Table 6. Other Health Care Service Utilisation Within One Year Prior of Entry into Long Term Care Facilities by Delirium or Dementia-related Hospitalisation Within 365 Days of Entry (Not Included in Table 1). 12](#_Toc200633749)

[Supplemental Table 7. Highlights of Individual, Medication, Health Care, System and Facility-Related Characteristics at Entry into Long-Term Care Facilities by Delirium or Dementia-Related Hospitalisation for the Model Training Sample. 15](#_Toc200633750)

[Supplemental Table 8. Highlights of Individual, Medication, Health Care, System and Facility-Related Characteristics at Entry into Long-Term Care Facilities by Delirium or Dementia-Related Hospitalisation for the Model Testing Sample. 18](#_Toc200633751)

[Comparison of model performance in the training and testing samples using Harrell’s C-index 21](#_Toc200633752)

[Supplemental Table 9. Model Performance Metrics Across Different Risk Thresholds in the Testing Sample. 22](#_Toc200633753)

[Supplemental Figure 1. Calibration Plot: Observed Vs. Predicted Risk of Delirium or Dementia-Related Hospitalisation Using the Training Sample. 23](#_Toc200633754)

[Supplemental Figure 2. Calibration Plot: Observed Vs. Predicted Risk of Delirium or Dementia-Related Hospitalisation Using Out-of-Sample Data. 24](#_Toc200633755)

[Supplemental Figure 3. Clinical Decision Curve Analysis (DCA) Using the Testing Sample: (A) base model based on age and sex; (B) final model. 25](#_Toc200633756)

[Supplemental Figure 4. The Predictive Performance of Delirium-Related Hospitalisation Only Risk Prediction Model (Out of Sample). AUC: Area Under the Receiver Operating Characteristics Curve; CI: Confidence Interval; SA: South Australia; VIC: Victoria; NSW: New South Wales; QLD: Queensland. 26](#_Toc200633757)

[TRIPOD+AI Checklist: Prediction Model Development and Validation. 27](#_Toc200633758)

# Supplementary Methods

Elastic net penalised regression approach

We fitted an elastic net penalised regression approach with a Fine and Gray model.^1^ Elastic net was selected due to its ability to manage the balance between model complexity and predictive accuracy, which is critical given the high-dimensional nature of our data and the potential for multicollinearity among predictors.^2,3^ This penalty shrinks the coefficients of less important variables toward zero while allowing for the selection of groups of correlated variables. In our study, we fitted an elastic net model to the training dataset, using the penalised regression framework to identify a subset of predictors of delirium or dementia-related hospitalisation. Variables with non-zero coefficients in the final model were selected for inclusion in the risk prediction model, ensuring a balance between predictive performance and model simplicity. The elastic net model requires two hyperparameters: the mixing parameter (α) and the regularization strength (λ). In this study, the tuning of hyperparameters was done using semi-manual approach, which involved exploring a range of hyperparameters to determine the optimal α and λ values through a grid search over a range of α (e.g., 0.1 to 1.0) and λ values (setting the penalty weight to 0 for age and sex, min = 0.002 and max = 0.0125), ensuring robust model performance.

Sample size

Given that our risk prediction model leveraged enormous amount of routinely collected national data from both the aged care and healthcare sectors, incorporating specific LTCF factors, and large enough to detect rare events, we did not calculate a priori sample size, as a convenience sample was used. However, using the pmsampsize R package^4^, for an approximately potential predictors of 458 variable, with a total of 632 levels, and assuming an event rate of 5.2% for time to dementia or delirium-related hospitalisation), the minimum required sample size was estimated to be approximately 19,600 (including at least 1,019 events). This requirement is well within the size of our study cohort.

# Supplemental Table 1. International Classification of Diseases, 10th Revision, Australian Modification (ICD-10-AM) Codes to Identify Delirium or Dementia-related Hospitalisation.

| **Description** | **ICD-10-AM** |
| --- | --- |
| Other symptoms and signs involving cognitive functions and awareness | R41 |
| Disorientation, unspecified | R41.0 |
| Other and unspecified symptoms and signs involving cognitive functions and awareness | R41.8 |
| Alzheimer’s disease | G30* |
| Lewy body disease | G31.3 |
| Dementia in Alzheimer’s disease | F00* |
| Vascular dementia | F01* |
| Dementia in other diseases classified elsewhere | F02* |
| Unspecified dementia | F03* |
| Delirium, not induced by alcohol and other psychoactive substances | F05* |

ICD-10-AM: International Classification of Diseases, 10th Revision, Australian Modification.

# Supplemental Table 2. Individual Characteristics at Entry into Long Term Care Facilities by Delirium or Dementia-related Hospitalisation Status Within 365 Days of Entry (Not Included in Table 1).

| **Characteristics** | **Total**  **N= 207343 (100.0%)** | **Delirium or dementia-related hospitalisation within 365 days**  **N=10709(5.2%)** | **No delirium or dementia-related hospitalisation within 365 days**  **N=196634(94.8%)** |
| --- | --- | --- | --- |
| **Preferred Language (English vs other)^*^** | 179797(86.7) | 8874(82.9) | 170923(86.9) |
| **Born in Australia^*^** | 131871(63.6) | 6200(57.9) | 125671(63.9) |
| **Marital Status** | 11744(5.7) | 561(5.2) | 11183(5.7) |
| Single | 87729(42.3) | 5278(49.3) | 82451(41.9) |
| Married | 88927(42.9) | 3782(35.3) | 85145(43.3) |
| Widowed | 18943(9.1) | 1088(10.2) | 17855(9.1) |
| Divorced, separated, or not determined | 11744(5.7) | 561(5.2) | 11183(5.7) |
| **Living Arrangements*** |  |  |  |
| Lives alone | 82999(40.0) | 3777(35.3) | 79222(40.3) |
| Lives with family | 115230(55.6) | 6460(60.3) | 108770(55.3) |
| Lives with others | 4616(2.2) | 214(2.0) | 4402(2.2) |
| **Carer Availability*** |  |  |  |
| No carer | 19492(9.4) | 999(9.3) | 18493(9.4) |
| **SEIFA Index of Relative Socio-Economic Advantage and Disadvantage*** |  |  |  |
| Q1 (greater disadvantage) | 37738(18.2) | 1915(17.9) | 35823(18.2) |
| Q2 | 35727(17.2) | 1700(15.9) | 34027(17.3) |
| Q3 | 37979(18.3) | 2044(19.1) | 35935(18.3) |
| Q4 | 39733(19.2) | 2309(21.6) | 37424(19.0) |
| Q5 (lower disadvantage) | 54133(26.1) | 2627(24.5) | 51506(26.2) |
| **SEIFA Index of Education and Occupation*** |  |  |  |
| Q1 (greater disadvantage) | 38552(18.6) | 1977(18.5) | 36575(18.6) |
| Q2 | 34732(16.8) | 1737(16.2) | 32995(16.8) |
| Q3 | 37749(18.2) | 2051(19.2) | 35698(18.2) |
| Q4 | 37964(18.3) | 2118(19.8) | 35846(18.2) |
| Q5 (lower disadvantage) | 56314(27.2) | 2712(25.3) | 53602(27.3) |
| **Health conditions^†^** |  |  |  |
| Malnutrition | 13126(6.3) | 627(5.9) | 12499(6.4) |
| Colorectal (bowel) cancer | 7673(3.7) | 331(3.1) | 7342(3.7) |
| Skin cancer | 10750(5.2) | 518(4.8) | 10232(5.2) |
| Breast cancer | 6885(3.3) | 278(2.6) | 6607(3.4) |
| Prostate cancer | 9492(4.6) | 560(5.2) | 8932(4.5) |
| Other malignant tumours | 7051(3.4) | 295(2.8) | 6756(3.4) |
| Other neoplasms | 4760(2.3) | 240(2.2) | 4520(2.3) |
| Anaemia | 12036(5.8) | 524(4.9) | 11512(5.9) |
| Other diseases of blood & blood forming organs & immune mechanism N.O.S or N.E.C | 5270(2.5) | 270(2.5) | 5000(2.5) |
| Disorders of the thyroid gland | 19640(9.5) | 874(8.2) | 18766(9.5) |
| Diabetes mellitus—Type 1 | 6502(3.1) | 376(3.5) | 6126(3.1) |
| Diabetes mellitus—Type 2 | 43170(20.8) | 2578(24.1) | 40592(20.6) |
| High cholesterol | 46953(22.6) | 2605(24.3) | 44348(22.6) |
| Other endocrine, nutritional & metabolic disorders | 11521(5.6) | 581(5.4) | 10940(5.6) |
| Dementia in Alzheimer’s disease | 148279(71.5) | 7591(70.9) | 140688(71.5) |
| Dementia in Alzheimer’s disease with late onset (>65 yrs) | 15866(7.7) | 782(7.3) | 15084(7.7) |
| Dementia in Alzheimer’s disease, atypical or mixed type | 7732(3.7) | 490(4.6) | 7242(3.7) |
| Dementia in Alzheimer’s disease (not further specified) | 14145(6.8) | 777(7.3) | 13368(6.8) |
| Vascular dementia | 32583(15.7) | 1917(17.9) | 30666(15.6) |
| Vascular dementia—unspecified | 5793(2.8) | 309(2.9) | 5484(2.8) |
| Dementia in other diseases classified elsewhere | 11333(5.5) | 638(6.0) | 10695(5.4) |
| Other dementia | 30563(14.7) | 1688(15.8) | 28875(14.7) |
| Unspecified dementia | 31820(15.3) | 1477(13.8) | 30343(15.4) |
| Depression/Mood affective disorders | 81588(39.3) | 4612(43.1) | 76976(39.1) |
| Neurotic, stress-related & somatoform disorders | 29864(14.4) | 1644(15.4) | 28220(14.4) |
| Phobic & anxiety disorders | 18543(8.9) | 953(8.9) | 17590(8.9) |
| Transient cerebral ischaemic attacks (T.I.A.s) | 14899(7.2) | 751(7.0) | 14148(7.2) |
| Other diseases of the nervous system | 16715(8.1) | 938(8.8) | 15777(8.0) |
| Cataracts | 19981(9.6) | 949(8.9) | 19032(9.7) |
| Glaucoma | 16864(8.1) | 845(7.9) | 16019(8.1) |
| Blindness (one or both eyes, one eye & low vision in other eye) | 8898(4.3) | 426(4.0) | 8472(4.3) |
| Poor vision (low vision one or both eyes, unspecified visual loss) | 25622(12.4) | 1183(11.0) | 24439(12.4) |
| Other diseases of the eye & adnexa | 9644(4.7) | 452(4.2) | 9192(4.7) |
| Ménière’s disease | 4536(2.2) | 223(2.1) | 4313(2.2) |
| Deafness/hearing loss | 43796(21.1) | 2172(20.3) | 41624(21.2) |
| Heart Disease | 51573(24.9) | 2683(25.1) | 48890(24.9) |
| Angina | 6538(3.2) | 324(3.0) | 6214(3.2) |
| Myocardial infarction (heart attack) | 11667(5.6) | 623(5.8) | 11044(5.6) |
| Congestive heart failure (congestive heart disease) | 19012(9.2) | 893(8.3) | 18119(9.2) |
| Other heart diseases | 28060(13.5) | 1468(13.7) | 26592(13.5) |
| Cerebrovascular disease | 9583(4.6) | 432(4.0) | 9151(4.7) |
| Cerebral infarction | 4336(2.1) | 224(2.1) | 4112(2.1) |
| Stroke (CVA)—cerebrovascular accident unspecified | 30286(14.6) | 1529(14.3) | 28757(14.6) |
| Other cerebrovascular diseases | 4826(2.3) | 267(2.5) | 4559(2.3) |
| Other diseases of the circulatory system | 4681(2.3) | 242(2.3) | 4439(2.3) |
| Hypertension (high blood pressure) | 119826(57.8) | 6086(56.8) | 113740(57.8) |
| Hypotension (low blood pressure) | 7491(3.6) | 397(3.7) | 7094(3.6) |
| Other diseases of the circulatory system | 20002(9.6) | 956(8.9) | 19046(9.7) |
| Influenza & pneumonia | 6171(3.0) | 286(2.7) | 5885(3.0) |
| Chronic lower respiratory diseases | 33277(16.0) | 1643(15.3) | 31634(16.1) |
| Other diseases of the respiratory system | 5345(2.6) | 254(2.4) | 5091(2.6) |
| Diseases of the intestine | 38738(18.7) | 1960(18.3) | 36778(18.7) |
| Other diseases of the digestive system | 37059(17.9) | 1874(17.5) | 35185(17.9) |
| Rheumatoid arthritis | 6200(3.0) | 293(2.7) | 5907(3.0) |
| Other arthritis & related disorders | 105289(50.8) | 5282(49.3) | 100007(50.9) |
| Back problems—dorsopathies | 22768(11.0) | 1167(10.9) | 21601(11.0) |
| Other disorders of the musculoskeletal system & connective tissue | 14589(7.0) | 706(6.6) | 13883(7.1) |
| Kidney & urinary system (bladder) disorders | 28984(14.0) | 1541(14.4) | 27443(14.0) |
| Urinary tract infection | 20664(10.0) | 957(8.9) | 19707(10.0) |
| Other diseases of the genitourinary system | 17178(8.3) | 1009(9.4) | 16169(8.2) |
| Injuries to arm/hand/shoulder | 7165(3.5) | 403(3.8) | 6762(3.4) |
| Injuries to leg/knee/foot/ankle/hip | 9301(4.5) | 457(4.3) | 8844(4.5) |
| Fracture of rib(s), sternum & thoracic spine | 4579(2.2) | 194(1.8) | 4385(2.2) |
| Fracture of lumbar spine & pelvis | 8499(4.1) | 374(3.5) | 8125(4.1) |
| Fracture of shoulder, upper arm & forearm | 6811(3.3) | 295(2.8) | 6516(3.3) |
| Fracture of femur | 17320(8.4) | 662(6.2) | 16658(8.5) |
| Pain | 49259(23.8) | 2559(23.9) | 46700(23.7) |
| Dysphagia | 7794(3.8) | 347(3.2) | 7447(3.8) |
| Abnormal involuntary movements | 4796(2.3) | 207(1.9) | 4589(2.3) |
| Amnesia | 62984(30.4) | 3018(28.2) | 59966(30.5) |
| Dizziness & giddiness | 9755(4.7) | 450(4.2) | 9305(4.7) |
| Speech & voice disturbances | 7268(3.5) | 360(3.4) | 6908(3.5) |
| Malaise & fatigue | 13784(6.6) | 546(5.1) | 13238(6.7) |
| Oedema N.E.C | 13699(6.6) | 685(6.4) | 13014(6.6) |
| Symptoms & signs concerning food & fluid intake | 14064(6.8) | 563(5.3) | 13501(6.9) |
| Has other health condition not elsewhere specified | 22344(10.8) | 1166(10.9) | 21178(10.8) |
| No formal diagnosis available | 5361(2.6) | 342(3.2) | 5019(2.6) |

SEIFA: Socio-Economic Indexes for Areas. N.O.S: Not otherwise specified; N.E.C: Not elsewhere classifiable. *Missing data: living arrangement (N=4498); Carer Availability (N=4598); SEIFA Index of Relative Socio-Economic Advantage and Disadvantage (N= 2033); SEIFA Index of Education and Occupation (N=2032). ^†^Only health conditions with prevalence of ≥2% are presented.

# Supplemental Table 3. Individual and Facility-related Characteristics at Entry into Long Term Care Facilities from Aged Care Funding Instrument Assessment^*^ by Delirium or Dementia-related Hospitalisation Status Within 365 Days of Entry (Not Included in Table 1).

| **Characteristics^*^** | **Total**  **N= 207343 (100.0%)** | **Delirium or dementia-related hospitalisation within 365 days**  **N=10709(5.2%)** | **No delirium or dementia-related hospitalisation within 365 days**  **N=196634(94.8%)** |
| --- | --- | --- | --- |
| **Provider Type** |  |  |  |
| Government | 9932(4.8) | 323(3.0) | 9609(4.9) |
| Private | 90626(43.7) | 5047(47.1) | 85579(43.5) |
| Not-for-profit | 106785(51.5) | 5339(49.9) | 101446(51.6) |
| **Location** |  |  |  |
| New South Wales | 82922(40.0) | 3964(37.0) | 78958(40.2) |
| South Australia | 22708(11.0) | 1125(10.5) | 21583(11.0) |
| Victoria | 60039(29.0) | 3352(31.3) | 56687(28.8) |
| Queensland | 41674(20.1) | 2268(21.2) | 39406(20.0) |
| **Complex Health Care Needs Level** |  |  |  |
| No or minimal impairment | 18142 (8.7) | 731 (6.8) | 17411 (8.9) |
| Mild impairment | 66526 (32.1) | 3462 (32.3) | 63064 (32.1) |
| Moderate impairment | 57482 (27.7) | 3158 (29.5) | 54324 (27.6) |
| High impairment | 63981 (30.9) | 3305 (30.9) | 60676 (30.9) |
| **Nutrition rating** |  |  |  |
| D worst | 42234(20.4) | 2278(21.3) | 39956(20.3) |
| C | 107258(51.7) | 5846(54.6) | 101412(51.6) |
| B | 46106(22.2) | 2134(19.9) | 43972(22.4) |
| A best | 10533(5.1) | 398(3.7) | 10135(5.2) |
| **Hygiene rating** |  |  |  |
| D worst | 162554(78.4) | 8780(82.0) | 153774(78.2) |
| C | 25607(12.4) | 1133(10.6) | 24474(12.4) |
| B | 16744(8.1) | 692(6.5) | 16052(8.2) |
| A best | 1226(0.6) | 51(0.5) | 1175(0.6) |
| **Toileting rating** |  |  |  |
| D worst | 129625(62.5) | 6942(64.8) | 122683(62.4) |
| C | 30611(14.8) | 1700(15.9) | 28911(14.7) |
| B | 32982(15.9) | 1542(14.4) | 31440(16.0) |
| A best | 12913(6.2) | 472(4.4) | 12441(6.3) |
| **Continence rating** |  |  |  |
| D worst | 147859(71.3) | 7906(73.8) | 139953(71.2) |
| C | 12490(6.0) | 606(5.7) | 11884(6.0) |
| B | 8692(4.2) | 440(4.1) | 8252(4.2) |
| A best | 37090(17.9) | 1704(15.9) | 35386(18.0) |
| **Cognitive Rating** |  |  |  |
| A (best) | 7544 (3.6) | 267 (2.5) | 7277 (3.7) |
| B | 43090 (20.8) | 1546 (14.4) | 41544 (21.1) |
| C | 80565 (38.9) | 3914 (36.5) | 76651 (39.0) |
| D (worst) | 74932 (36.1) | 4929 (46.0) | 70003 (35.6) |
| **Depression and dysthymia rating** |  |  |  |
| D worst | 23219(11.2) | 1482(13.8) | 21737(11.1) |
| C | 26011(12.5) | 1471(13.7) | 24540(12.5) |
| B | 62651(30.2) | 3248(30.3) | 59403(30.2) |
| A best | 94250(45.5) | 4455(41.6) | 89795(45.7) |
| **Medication assistance rating** |  |  |  |
| D worst | 47255(22.8) | 2700(25.2) | 44555(22.7) |
| C | 59952(28.9) | 2935(27.4) | 57017(29.0) |
| B | 96396(46.5) | 4946(46.2) | 91450(46.5) |
| A best | 2528(1.2) | 75(0.7) | 2453(1.2) |

**^*^**Missing data N(%): 1212(0.6).

# Supplemental Table 4. Medication-related *C*haracteristics in the 90 Days Prior to Entry into Long Term Care Facilities by Delirium or Dementia-related Hospitalisation Status Within 365 Days of Entry (Not Included in Table 1).

| **Characteristics*** | | **Total**  **N= 207343 (100.0%)** | **Delirium or dementia-related hospitalisation within 365 days**  **N=10709(5.2%)** | **No delirium or dementia-related hospitalisation within 365 days**  **N=196634 (94.8%)** |
| --- | --- | --- | --- | --- |
| **ATC code** | **Chemical subgroup name** |  |  |  |
| A02BA | H2-receptor antagonists | 5109(2.5) | 230(2.1) | 4879(2.5) |
| A02BC | Proton pump inhibitors | 75239(36.3) | 3981(37.2) | 71258(36.2) |
| A06AD | Osmotically acting laxatives | 34068(16.4) | 1943(18.1) | 32125(16.3) |
| A10BA | Biguanides | 18463(8.9) | 1166(10.9) | 17297(8.8) |
| A10BB | Sulfonylureas | 12208(5.9) | 722(6.7) | 11486(5.8) |
| A12BA | Potassium | 9028(4.4) | 408(3.8) | 8620(4.4) |
| B01AA | Vitamin K antagonists | 14255(6.9) | 779(7.3) | 13476(6.9) |
| B01AB | Heparin group | 9656(4.7) | 402(3.8) | 9254(4.7) |
| B01AC | Platelet aggregation inhibitors excl. heparin | 51285(24.7) | 2689(25.1) | 48596(24.7) |
| B03BA | Vitamin B12 (cyanocobalamin and analogues) | 8221(4.0) | 385(3.6) | 7836(4.0) |
| B03BB | Folic acid and derivatives | 4350(2.1) | 187(1.7) | 4163(2.1) |
| C01AA | Digitalis glycosides | 11928(5.8) | 571(5.3) | 11357(5.8) |
| C01BD | Antiarrhythmics, class III | 6004(2.9) | 302(2.8) | 5702(2.9) |
| C01DA | Organic nitrates | 14799(7.1) | 793(7.4) | 14006(7.1) |
| C03CA | Sulfonamides, plain | 34847(16.8) | 1667(15.6) | 33180(16.9) |
| C03DA | Aldosterone antagonists | 6716(3.2) | 294(2.7) | 6422(3.3) |
| C07AB | Beta blocking agents, selective | 42638(20.6) | 2357(22.0) | 40281(20.5) |
| C08CA | Dihydropyridine derivatives | 28325(13.7) | 1413(13.2) | 26912(13.7) |
| C08DB | Benzothiazepine derivatives | 4526(2.2) | 198(1.8) | 4328(2.2) |
| C09AA | ACE inhibitors, plain | 42462(20.5) | 2100(19.6) | 40362(20.5) |
| C09CA | Angiotensin II antagonists, plain | 32517(15.7) | 1753(16.4) | 30764(15.6) |
| C09DA | Angiotensin II antagonists and diuretics | 9237(4.5) | 469(4.4) | 8768(4.5) |
| C10AA | HMG CoA reductase inhibitors | 76677(37.0) | 4175(39.0) | 72502(36.9) |
| D07AC | Corticosteroids, potent (group III) | 9912(4.8) | 521(4.9) | 9391(4.8) |
| G04BD | Urinary antispasmodics | 4841(2.3) | 256(2.4) | 4585(2.3) |
| H02AB | Glucocorticoids | 12518(6.0) | 600(5.6) | 11918(6.1) |
| H03AA | Thyroid hormones | 13320(6.4) | 606(5.7) | 12714(6.5) |
| J01AA | Tetracyclines | 5088(2.5) | 275(2.6) | 4813(2.4) |
| J01CA | Penicillins with extended spectrum | 9910(4.8) | 508(4.7) | 9402(4.8) |
| J01CF | Beta-lactamase resistant penicillins | 4313(2.1) | 214(2.0) | 4099(2.1) |
| J01CR | Combinations of penicillins, incl. beta-lactamase inhibitors | 14368(6.9) | 739(6.9) | 13629(6.9) |
| J01DB | First-generation cephalosporins | 31474(15.2) | 1560(14.6) | 29914(15.2) |
| J01EA | Trimethoprim and derivatives | 15121(7.3) | 773(7.2) | 14348(7.3) |
| J01FA | Macrolides | 7746(3.7) | 389(3.6) | 7357(3.7) |
| M01AC | Oxicams | 4908(2.4) | 243(2.3) | 4665(2.4) |
| M04AA | Preparations inhibiting uric acid production | 8059(3.9) | 477(4.5) | 7582(3.9) |
| M05BA | Bisphosphonates | 11127(5.4) | 471(4.4) | 10656(5.4) |
| M05BB | Bisphosphonates, combinations | 10037(4.8) | 400(3.7) | 9637(4.9) |
| M05BX | Other drugs affecting bone structure and mineralization | 7641(3.7) | 334(3.1) | 7307(3.7) |
| N02AA | Natural opium alkaloids | 24179(11.7) | 1168(10.9) | 23011(11.7) |
| N02AE | Oripavine derivatives | 10155(4.9) | 492(4.6) | 9663(4.9) |
| N02AJ | Opioids in combination with non-opioid analgesics | 6542(3.2) | 327(3.1) | 6215(3.2) |
| N02AX | Other opioids | 4728(2.3) | 244(2.3) | 4484(2.3) |
| N02BE | Anilides | 65799(31.7) | 3324(31.0) | 62475(31.8) |
| N02BF | N02BF | 5550(2.7) | 357(3.3) | 5193(2.6) |
| N04BA | Dopa and dopa derivatives | 12835(6.2) | 728(6.8) | 12107(6.2) |
| N05AD | Butyrophenone derivatives | 5892(2.8) | 446(4.2) | 5446(2.8) |
| N05AX | Other antipsychotics | 9921(4.8) | 709(6.6) | 9212(4.7) |
| N05CD | Benzodiazepine derivatives | 21723(10.5) | 1287(12.0) | 20436(10.4) |
| N06AA | Non-selective monoamine reuptake inhibitors | 8469(4.1) | 402(3.8) | 8067(4.1) |
| N06AB | Selective serotonin reuptake inhibitors | 36735(17.7) | 2141(20.0) | 34594(17.6) |
| N06AX | Other antidepressants | 26396(12.7) | 1660(15.5) | 24736(12.6) |
| R03AC | Selective beta-2-adrenoreceptor agonists | 14175(6.8) | 687(6.4) | 13488(6.9) |
| R03AK | Adrenergics in combination with corticosteroids or other drugs, excl. anticholinergics | 14194(6.8) | 699(6.5) | 13495(6.9) |
| R03BB | Anticholinergics | 12329(5.9) | 594(5.5) | 11735(6.0) |
| S01AA | Antibiotics | 6195(3.0) | 286(2.7) | 5909(3.0) |
| S01EC | Carbonic anhydrase inhibitors | 4068(2.0) | 229(2.1) | 3839(2.0) |
| S01EE | Prostaglandin analogues1) | 16009(7.7) | 872(8.1) | 15137(7.7) |
| S01XA | Other ophthalmologicals | 14921(7.2) | 751(7.0) | 14170(7.2) |

*Only medicine classes with prevalence of ≥2% are presented.

# Supplemental Table 5. Hospital-related Health Care Utilisation Within One Year Prior of Entry into Long Term Care Facilities by Delirium or Dementia-related Hospitalisation Within 365 Days of Entry (Not Included in Table 1).

| **Characteristics** | **Total**  **N= 207343 (100.0%)** | **Delirium or dementia-related hospitalisation within 365 days**  **N=1070(5.2%)** | **No delirium or dementia-related hospitalisation within 365 days**  **N=196634(94.8%)** |
| --- | --- | --- | --- |
| **Total length of stay in hospital** |  |  |  |
| 0 | 58019(28.0) | 2605(24.3) | 55414(28.2) |
| 1-30 days | 76387(36.8) | 3958(37.0) | 72429(36.8) |
| 31-90 days | 59133(28.5) | 3272(30.6) | 55861(28.4) |
| 90+ days | 13804(6.7) | 874(8.2) | 12930(6.6) |
| **Days since last hospitalisation separation** |  |  |  |
| 0-20 | 12821(6.2) | 669(6.2) | 12152(6.2) |
| 21-100 | 53610(25.9) | 2746(25.6) | 50864(25.9) |
| 101-150 | 17043(8.2) | 929(8.7) | 16114(8.2) |
| 151-364 | 59303(28.6) | 3485(32.5) | 55818(28.4) |
| >=365 | 64566(31.1) | 2880(26.9) | 61686(31.4) |
| **Number of Potentially Preventable Hospitalisations (PPH)** |  |  |  |
| 0 | 168198 (81.1) | 8604 (80.3) | 159594 (81.2) |
| 1 | 25621 (12.4) | 1383 (12.9) | 24238 (12.3) |
| 2-4 | 12387 (6.0) | 656 (6.1) | 11731 (6.0) |
| ≥5 | 1137 (0.5) | 66 (0.6) | 1071 (0.5) |
| **Total length of stay in hospital during PPH** |  |  |  |
| 0 | 170838(82.4) | 8748(81.7) | 162090(82.4) |
| 1-30 days | 31111(15.0) | 1683(15.7) | 29428(15.0) |
| 31-90 days | 5023(2.4) | 254(2.4) | 4769(2.4) |
| 90+ days | 371(0.2) | 24(0.2) | 347(0.2) |
| **Days since last hospitalisation separation** |  |  |  |
| 0-20 | 3687(1.8) | 160(1.5) | 3527(1.8) |
| 21-100 | 15391(7.4) | 801(7.5) | 14590(7.4) |
| 101-150 | 5020(2.4) | 287(2.7) | 4733(2.4) |
| 151-364 | 14978(7.2) | 853(8.0) | 14125(7.2) |
| >=365 | 168267(81.2) | 8608(80.4) | 159659(81.2) |

# Supplemental Table 6. Other Health Care Service Utilisation Within One Year Prior of Entry into Long Term Care Facilities by Delirium or Dementia-related Hospitalisation Within 365 Days of Entry (Not Included in Table 1).

| **Characteristics** | **Total**  **N= 207343 (100.0%)** | **Delirium or dementia-related hospitalisation within 365 days**  **N=10709(5.2%)** | **No delirium or dementia-related hospitalisation within 365 days**  **N=196634(94.8%)** |
| --- | --- | --- | --- |
| **General Practitioner (GP) After-Hours Attendances to which No Other Item Applies** |  |  |  |
| 0 | 150421(72.5) | 7227(67.5) | 143194(72.8) |
| 1 | 29206(14.1) | 1707(15.9) | 27499(14.0) |
| 2-4 | 20578(9.9) | 1272(11.9) | 19306(9.8) |
| ≥5 | 7138(3.4) | 503(4.7) | 6635(3.4) |
| **Other Non-Referred After-Hours Attendances to which No Other Item Applies** |  |  |  |
| 0 | 202484(97.7) | 10456(97.6) | 192028(97.7) |
| 1 | 3886(1.9) | 199(1.9) | 3687(1.9) |
| 2-4 | 887(0.4) | 48(0.4) | 839(0.4) |
| ≥5 | 86(0.0) | 6(0.1) | 80(0.0) |
| **Urgent Attendance After Hours** |  |  |  |
| 0 | 168641(81.3) | 8405(78.5) | 160236(81.5) |
| 1 | 25182(12.1) | 1427(13.3) | 23755(12.1) |
| 2-4 | 11907(5.7) | 780(7.3) | 11127(5.7) |
| ≥5 | 1613(0.8) | 97(0.9) | 1516(0.8) |
| **GP Mental Health Treatment** |  |  |  |
| 0 | 197953(95.5) | 10045(93.8) | 187908(95.6) |
| 1 | 6216(3.0) | 425(4.0) | 5791(2.9) |
| 2-4 | 2584(1.2) | 189(1.8) | 2395(1.2) |
| ≥5 | 590(0.3) | 50(0.5) | 540(0.3) |
| **GP Attendance Associated with PIP Incentive Payments** |  |  |  |
| 0 | 200361(96.6) | 10297(96.2) | 190064(96.7) |
| ≥1 | 6916(3.3) | 411(3.8) | 6505(3.3) |
| **Health Assessments** |  |  |  |
| 0 | 143594(69.3) | 7444(69.5) | 136150(69.2) |
| 1 | 59530(28.7) | 3042(28.4) | 56488(28.7) |
| ≥2 | 4219(2.0) | 223(2.1) | 3996(2.0) |
| **Number of GP Management Plans, Team Care Arrangements, Multidisciplinary Care Plans** |  |  |  |
| 0 | 106800(51.5) | 5282(49.3) | 101518(51.6) |
| 1 | 20034(9.7) | 957(8.9) | 19077(9.7) |
| ≥2 | 80509(38.8) | 4470(41.7) | 76039(38.7) |
| **Medication Management Reviews** |  |  |  |
| 0 | 197036(95.0) | 10131(94.6) | 186905(95.1) |
| 1 | 10169(4.9) | 566(5.3) | 9603(4.9) |
| ≥2 | 138(0.1) | 12(0.1) | 126(0.1) |
| **Optometrical Services** |  |  |  |
| 0 | 141883(68.4) | 7061(65.9) | 134822(68.6) |
| 1 | 50005(24.1) | 2752(25.7) | 47253(24.0) |
| 2-4 | 14742(7.1) | 850(7.9) | 13892(7.1) |
| ≥5 | 713(0.3) | 46(0.4) | 667(0.3) |
| **Nurse Practitioners** |  |  |  |
| 0 | 205293(99.0) | 10606(99.0) | 194687(99.0) |
| 1 | 926(0.4) | 50(0.5) | 876(0.4) |
| 2-4 | 722(0.3) | 35(0.3) | 687(0.3) |
| ≥5 | 402(0.2) | 18(0.2) | 384(0.2) |
| **Number of Allied Health Services** |  |  |  |
| 0 | 143920(69.4) | 7064(66.0) | 136856(69.6) |
| 1 | 12471(6.0) | 689(6.4) | 11782(6.0) |
| 2-4 | 37327(18.0) | 2140(20.0) | 35187(17.9) |
| ≥5 | 13625(6.6) | 816(7.6) | 12809(6.5) |
| **Focussed Psychological Strategies (Allied Mental Health)** |  |  |  |
| 0 | 205810(99.3) | 10593(98.9) | 195217(99.3) |
| 1 | 416(0.2) | 30(0.3) | 386(0.2) |
| 2-4 | 641(0.3) | 41(0.4) | 600(0.3) |
| ≥5 | 476(0.2) | 45(0.4) | 431(0.2) |
| **Medical Practitioner (Emergency Physician) Attendances to which No Other Item Applies** |  |  |  |
| 0 | 200916(96.9) | 10372(96.9) | 190544(96.9) |
| 1 | 4879(2.4) | 238(2.2) | 4641(2.4) |
| ≥2 | 1548(0.7) | 99(0.9) | 1449(0.7) |
| **Neurosurgery Attendances to which No Other Item Applies** |  |  |  |
| 0 | 205341(99.0) | 10613(99.1) | 194728(99.0) |
| 1 | 962(0.5) | 48(0.4) | 914(0.5) |
| ≥2 | 1040(0.5) | 48(0.4) | 992(0.5) |
| **Pain and Palliative Medicine** |  |  |  |
| 0 | 205901(99.3) | 10662(99.6) | 195239(99.3) |
| 1 | 579(0.3) | 18(0.2) | 561(0.3) |
| 2-4 | 468(0.2) | 18(0.2) | 450(0.2) |
| ≥5 | 395(0.2) | 11(0.1) | 384(0.2) |
| **Specialist Attendances to which No Other Item Applies** |  |  |  |
| 0 | 126348(60.9) | 6445(60.2) | 119903(61.0) |
| 1-5 | 71774(34.6) | 3828(35.7) | 67946(34.6) |
| 6-15 | 8394(4.0) | 403(3.8) | 7991(4.1) |
| ≥16 | 827(0.4) | 33(0.3) | 794(0.4) |
| **Consultant Physician Attendances to which No Other Item Applies** |  |  |  |
| 0 | 104753(50.5) | 5115(47.8) | 99638(50.7) |
| 1-5 | 61442(29.6) | 3491(32.6) | 57951(29.5) |
| 6-15 | 20242(9.8) | 1137(10.6) | 19105(9.7) |
| ≥16 | 20906(10.1) | 966(9.0) | 19940(10.1) |
| **Consultant Psychiatrist Attendances to which No Other Item Applies** |  |  |  |
| 0 | 197246(95.1) | 10032(93.7) | 187214(95.2) |
| 1-5 | 8025(3.9) | 531(5.0) | 7494(3.8) |
| 6-15 | 1469(0.7) | 108(1.0) | 1361(0.7) |
| ≥16 | 603(0.3) | 38(0.4) | 565(0.3) |
| **Other Non-Referred Attendances to which No Other Item Applies** |  |  |  |
| 0 | 189127(91.2) | 9736(90.9) | 179391(91.2) |
| 1 | 9414(4.5) | 465(4.3) | 8949(4.6) |
| 2-4 | 5349(2.6) | 286(2.7) | 5063(2.6) |
| ≥5 | 3453(1.7) | 222(2.1) | 3231(1.6) |

GP: General Practitioner; PIP: Practice Incentives Program.

# Supplemental Table 7. Highlights of Individual, Medication, Health Care, System and Facility-Related Characteristics at Entry into Long-Term Care Facilities by Delirium or Dementia-Related Hospitalisation for the Model Training Sample.

| **Characteristics** | **Total**  **N= 165874(100.0%)** | **Delirium or dementia-related hospitalisation within 365 days***  **N= 8616(5.2%)** | **No delirium or dementia-related hospitalisation within 365 days**  **N= 157258(94.8%)** |
| --- | --- | --- | --- |
| **Age Group, years** |  |  |  |
| 65-74 | 18046(10.9) | 1358(15.8) | 16688(10.6) |
| 75-84 | 70319(42.4) | 3953(45.9) | 66366(42.2) |
| 85-94 | 70900(42.7) | 3100(36.0) | 67800(43.1) |
| ≥95 | 6609(4.0) | 205(2.4) | 6404(4.1) |
| **Sex** |  |  |  |
| Men | 66058(39.8) | 4577(53.1) | 61481(39.1) |
| Women | 99816(60.2) | 4039(46.9) | 95777(60.1) |
| **Facility Remoteness** |  |  |  |
| Major cities | 119420(72.0) | 6810(79.0) | 112610(71.6) |
| Inner regional | 34581(20.8) | 1346(15.6) | 33235(21.1) |
| Outer regional | 10972(6.6) | 427(5.0) | 10545(6.7) |
| Remote or very remote | 512(0.3) | 19(0.2) | 493(0.3) |
| **SEIFA Index of Economic Resources** |  |  |  |
| Q1 (greater disadvantage) | 41501(25.0) | 2219(25.8) | 39282(25.0) |
| Q2 | 34768(21.0) | 1620(18.8) | 33148(21.1) |
| Q3 | 36018(21.7) | 1921(22.3) | 34097(21.7) |
| Q4 | 28771(17.3) | 1619(18.8) | 27152(17.3) |
| Q5 (lower disadvantage) | 23171(14.0) | 1144(13.3) | 22027(14.0) |
| **Health Conditions**^†^ |  |  |  |
| Incontinence (urinary or bowel) | 68479(41.3) | 3506(40.7) | 64973(41.3) |
| History of falls | 55090(33.2) | 2702(31.4) | 52388(33.3) |
| Abnormalities of gait & mobility | 48707(29.4) | 2167(25.2) | 46540(29.6) |
| Osteoporosis | 37680(22.7) | 1581(18.3) | 36099(23.0) |
| History of fractures | 36757(22.2) | 1509(17.5) | 35248(22.4) |
| Type 2 diabetes mellitus | 41388(25.0) | 2456(28.5) | 38932(24.8) |
| Psychoses | 34184(20.6) | 2367(27.5) | 31817(20.2) |
| Disorientation | 24778(14.9) | 1472(17.1) | 23306(14.8) |
| Acute and chronic ischaemic heart disease | 23900(14.4) | 1379(16.0) | 22521(14.3) |
| Parkinson’s disease | 12461(7.5) | 713(8.3) | 11748(7.5) |
| History of delirium | 12335(7.4) | 984(11.4) | 11351(7.2) |
| Breathing difficulties/shortness of breath | 6620(4.0) | 287(3.3) | 6333(4.0) |
| Restlessness & agitation | 7557(4.6) | 661(7.7) | 6896(4.4) |
| Other mental & behavioural disorders | 3782(2.3) | 295(3.4) | 3487(2.2) |
| Epilepsy | 3732(2.2) | 230(2.7) | 3502(2.2) |
| Irritability & anger | 3281(2.0) | 267(3.1) | 3014(1.9) |
| Mental and behavioural disorders due to alcohol and other psychoactive substance use | 2767(1.7) | 138(1.6) | 2629(1.7) |
| History of physical violence | 554(0.3) | 83(1.0) | 471(0.3) |
| **Rx-Risk-V Co-morbidity Category** |  |  |  |
| 0-1 | 21440(12.9) | 1027(11.9) | 20413(13.0) |
| 2-3 | 38838(23.4) | 1830(21.2) | 37008(23.5) |
| 4-5 | 47245(28.5) | 2384(27.7) | 44861(28.5) |
| 6-8 | 45624(27.5) | 2571(29.8) | 43053(27.4) |
| ≥9 | 12727(7.7) | 804(9.3) | 11923(7.6) |
| **Sedative Loading Rating** |  |  |  |
| 0 | 61550(37.1) | 2668(31.0) | 58882(37.4) |
| 1-2 | 64561(38.9) | 3321(38.5) | 61240(38.9) |
| ≥3 | 39763(24.0) | 2627(30.5) | 37136(23.6) |
| **Number of Unique Medications** |  |  |  |
| 0-4 | 61476(37.1) | 3054(35.4) | 58422(37.2) |
| 5-10 | 78695(47.4) | 4095(47.5) | 74600(47.4) |
| 11+ | 25703(15.5) | 1467(17.0) | 24236(15.4) |
| **Specific Medications**^‡^ |  |  |  |
| Benzodiazepine derivatives (N05BA) | 16297(9.8) | 1191(13.8) | 15106(9.6) |
| Diazepines, oxazepines, thiazepines and oxepines (N05AH) | 10852(6.5) | 874(10.1) | 9978(6.3) |
| Propulsives (A03FA) | 7959(4.8) | 315(3.7) | 7644(4.9) |
| Direct factor Xa inhibitors (B01AF) | 6539(3.9) | 424(4.9) | 6115(3.9) |
| Fatty acid derivative antiepileptics (N03AG) | 5222(3.1) | 437(5.1) | 4785(3.0) |
| Dipeptidyl peptidase 4 (DPP-4) inhibitors (A10BH) | 2565(1.5) | 179(2.1) | 2386(1.5) |
| Other antiepileptics (N03AX) | 2534(1.5) | 181(2.1) | 2353(1.5) |
| Monoamine oxidase type B inhibitors (N04BD) | 658(0.4) | 54(0.6) | 604(0.4) |
| **Activities of Daily Living Level**^§^ |  |  |  |
| No or minimal impairment | 2369(1.4) | 84(1.0) | 2285(1.5) |
| Mild impairment | 33985(20.5) | 1559(18.1) | 32426(20.6) |
| Moderate impairment | 55697(33.6) | 3122(36.2) | 52575(33.4) |
| High impairment | 72866(43.9) | 3808(44.2) | 69058(43.9) |
| **Behavioural Daily Living Level**^§^ |  |  |  |
| No or minimal impairment | 5594(3.4) | 141(1.6) | 5453(3.5) |
| Mild impairment | 25028(15.1) | 831(9.6) | 24197(15.4) |
| Moderate impairment | 35458(21.4) | 1400(16.2) | 34058(21.7) |
| High impairment | 98837(59.6) | 6201(72.0) | 92636(58.9) |
| **Complex Health Care Rating**^§^ |  |  |  |
| A (best) | 39406(23.8) | 2197(25.5) | 37209(23.7) |
| B | 40817(24.6) | 1993(23.1) | 38824(24.7) |
| C | 47032(28.4) | 2507(29.1) | 44525(28.3) |
| D (worst) | 37662(22.7) | 1876(21.8) | 35786(22.8) |
| **Mobility Rating**^§¶^ |  |  |  |
| A (best) | 7782(4.7) | 359(4.2) | 7423(4.7) |
| B | 12631(7.6) | 623(7.2) | 12008(7.6) |
| C | 68330(41.2) | 3911(45.4) | 64419(41.0) |
| D (worst) | 76174(45.9) | 3680(42.7) | 72494(46.1) |
| **Wandering Rating^§¶^** |  |  |  |
| A (best) | 104918(63.3) | 4677(54.3) | 100241(63.7) |
| B | 15928(9.6) | 798(9.3) | 15130(9.6) |
| C | 10551(6.4) | 583(6.8) | 9968(6.3) |
| D (worst) | 33520(20.2) | 2515(29.2) | 31005(19.7) |
| **Verbal Behaviour Rating^§¶^** |  |  |  |
| A (best) | 27537(16.6) | 960(11.1) | 26577(16.9) |
| B | 25402(15.3) | 1188(13.8) | 24214(15.4) |
| C | 27624(16.7) | 1408(16.3) | 26216(16.7) |
| D (worst) | 84354(50.9) | 5017(58.2) | 79337(50.5) |
| **Physical Behaviour Rating^§¶^** |  |  |  |
| A (best) | 45094(27.2) | 1609(18.7) | 43485(27.7) |
| B | 25970(15.7) | 1314(15.3) | 24656(15.7) |
| C | 24089(14.5) | 1228(14.3) | 22861(14.5) |
| D (worst) | 69764(42.1) | 4422(51.3) | 65342(41.6) |
| **Number of Unplanned Hospitalisations^#^** |  |  |  |
| None | 51181(30.9) | 2317(26.9) | 48864(31.1) |
| 1 | 55758(33.6) | 2818(32.7) | 52940(33.7) |
| 2-4 | 51659(31.1) | 2959(34.3) | 48700(31.0) |
| ≥5 | 7276(4.4) | 522(6.1) | 6754(4.3) |
| **Number of ED Presentations^#^** |  |  |  |
| 0 | 53775(32.4) | 2230(25.9) | 51545(32.8) |
| 1 | 48571(29.3) | 2486(28.9) | 46085(29.3) |
| 2-4 | 52347(31.6) | 3085(35.8) | 49262(31.3) |
| ≥5 | 11181(6.7) | 815(9.5) | 10366(6.6) |
| **Number of GP Attendances^#^** |  |  |  |
| 0 | 5550(3.3) | 261(3.0) | 5289(3.4) |
| 1-5 | 43902(26.5) | 2035(23.6) | 41867(26.6) |
| 6-15 | 79679(48.0) | 4224(49.0) | 75455(48.0) |
| ≥16 | 36743(22.2) | 2096(24.3) | 34647(22.0) |
| **Number of Geriatric Medicine Physician Attendances^#^** |  |  |  |
| 0 | 147552(89.0) | 7509(87.2) | 140043(89.1) |
| 1 | 12120(7.3) | 721(8.4) | 11399(7.2) |
| ≥2 | 6202(3.7) | 386(4.5) | 5816(3.7) |

ED=Emergency Department. GP=General Practitioner. ^#^Delirium or dementia-related hospitalisation within 365 days after LTCF entry, defined as hospitalisation or ED presentation where dementia or delirium was recorded as the principal discharge diagnosis for the encounter. ^†^Conditions were ascertained using the aged care eligibility or entry into care assessments. ^‡^Medications at the chemical subgroup level (ATC 4th level, i.e. 5 digits). ^¶^A is least dependant and D is most dependant. ^§^Care needs of LTCF residents from entry into LTCF assessments. ^#^Ascertained in the 365 days prior to LTCF entry. Missing data N(%): Facility Remoteness: 389(0.2); SEIFA Index of Economic Resources: 1645(1.0).

# Supplemental Table 8. Highlights of Individual, Medication, Health Care, System and Facility-Related Characteristics at Entry into Long-Term Care Facilities by Delirium or Dementia-Related Hospitalisation for the Model Testing Sample.

| **Characteristics** | **Total**  **N= 41469(100.0)** | **Delirium or dementia-related hospitalisation within 365 days***  **N=** **2093(5.05%)** | **No delirium or dementia-related hospitalisation within 365 days**  **N= 39376(94.95%)** |
| --- | --- | --- | --- |
| **Age Group, years** |  |  |  |
| 65-74 | 4526(10.9) | 286(13.7) | 4240(10.8) |
| 75-84 | 17456(42.1) | 950(45.4) | 16506(41.9) |
| 85-94 | 17884(43.1) | 815(38.9) | 17069(43.3) |
| ≥95 | 1603(3.9) | 42(2.0) | 1561(4.0) |
| **Sex** |  |  |  |
| Men | 16459(39.7) | 1084(51.8) | 15375(39.0) |
| Women | 25010(60.3) | 1009(48.2) | 24001(61.0) |
| **Facility Remoteness** |  |  |  |
| Major cities | 29939(72.2) | 1669(79.7) | 28270(71.8) |
| Inner regional | 8600(20.7) | 302(14.4) | 8298(21.1) |
| Outer regional | 2699(6.5) | 111(5.3) | 2588(6.6) |
| Remote or very remote | 151(0.4) | 9(0.4) | 142(0.4) |
| **SEIFA Index of Economic Resources** |  |  |  |
| Q1 (greater disadvantage) | 10385(25.0) | 546(26.1) | 9839(25.0) |
| Q2 | 8763(21.1) | 418(20.0) | 8345(21.2) |
| Q3 | 8780(21.2) | 450(21.5) | 8330(21.2) |
| Q4 | 7333(17.7) | 388(18.5) | 6945(17.6) |
| Q5 (lower disadvantage) | 5820(14.0) | 270(12.9) | 5550(14.1) |
| **Health Conditions**^†^ |  |  |  |
| Incontinence (urinary or bowel) | 17220(41.5) | 877(41.9) | 16343(41.5) |
| History of falls | 13795(33.3) | 705(33.7) | 13090(33.2) |
| Abnormalities of gait & mobility | 12098(29.2) | 526(25.1) | 11572(29.4) |
| Osteoporosis | 9289(22.4) | 407(19.4) | 8882(22.6) |
| History of fractures | 9177(22.1) | 402(19.2) | 8775(22.3) |
| Type 2 diabetes mellitus | 10313(24.9) | 597(2093) | 9716(24.7) |
| Psychoses | 8469(20.4) | 586(28.0) | 7883(20.0) |
| Disorientation | 6163(14.9) | 365(17.4) | 5798(14.7) |
| Acute and chronic ischaemic heart disease | 6032(14.5) | 364(17.4) | 5668(14.4) |
| Parkinson’s disease | 3107(7.5) | 189(9.0) | 2918(7.4) |
| History of delirium | 3085(7.4) | 244(11.7) | 2841(7.2) |
| Breathing difficulties/shortness of breath | 1663(4.0) | 65(3.1) | 1598(4.1) |
| Restlessness & agitation | 1918(4.6) | 147(7.0) | 1771(4.5) |
| Other mental & behavioural disorders | 1011(2.4) | 81(3.9) | 930(2.4) |
| Epilepsy | 862(2.1) | 56(2.7) | 806(2.0) |
| Irritability & anger | HC_1721 | 840(2.0) | 66(3.2) |
| Mental and behavioural disorders due to alcohol and other psychoactive substance use | 687(1.7) | 34(1.6) | 653(1.7) |
| History of physical violence | 147(0.4) | 18(0.9) | 129(0.3) |
| **Rx-Risk-V Co-morbidity Category** |  |  |  |
| 0-1 | 5552(13.4) | 226(10.8) | 5326(13.5) |
| 2-3 | 9485(22.9) | 470(22.5) | 9015(22.9) |
| 4-5 | 11954(28.8) | 571(27.3) | 11383(28.9) |
| 6-8 | 11300(27.2) | 638(30.5) | 10662(27.1) |
| ≥9 | 3178(7.7) | 188(9.0) | 2990(7.6) |
| **Sedative Loading Rating** |  |  |  |
| 0 | 15291(36.9) | 647(30.9) | 14644(37.2) |
| 1-2 | 16178(39.0) | 810(38.7) | 15368(39.0) |
| ≥3 | 10000(24.1) | 636(30.4) | 9364(23.8) |
| **Number of Unique Medications** |  |  |  |
| 0-4 | 15464(37.3) | 719(34.4) | 14745(37.4) |
| 5-10 | 19613(47.3) | 1003(47.9) | 18610(47.3) |
| 11+ | 6392(15.4) | 371(17.7) | 6021(15.3) |
| **Specific Medications**^‡^ |  |  |  |
| Benzodiazepine derivatives (N05BA) | 4072(9.8) | 300(14.3) | 3772(9.6) |
| Diazepines, oxazepines, thiazepines and oxepines (N05AH) | 2686(6.5) | 217(10.4) | 2469(6.3) |
| Propulsives (A03FA) | 1933(4.7) | 90(4.3) | 1843(4.7) |
| Direct factor Xa inhibitors (B01AF) | 1667(4.0) | 106(5.1) | 1561(4.0) |
| Fatty acid derivative antiepileptics (N03AG) | 1291(3.1) | 81(3.9) | 1210(3.1) |
| Dipeptidyl peptidase 4 (DPP-4) inhibitors (A10BH) | 641(1.5) | 54(2.6) | 587(1.5) |
| Other antiepileptics (N03AX) | 625(1.5) | 50(2.4) | 575(1.5) |
| Monoamine oxidase type B inhibitors (N04BD) | 174(0.4) | 20(1.0) | 154(0.4) |
| **Activities of Daily Living Level**^§^ |  |  |  |
| No or minimal impairment | 576(1.4) | 19(0.9) | 557(1.4) |
| Mild impairment | 8418(20.3) | 352(16.8) | 8066(20.5) |
| Moderate impairment | 13874(33.5) | 780(37.3) | 13094(33.3) |
| High impairment | 18346(44.2) | 932(44.5) | 17414(44.2) |
| **Behavioural Daily Living Level**^§^ |  |  |  |
| No or minimal impairment | 1369(3.3) | 39(1.9) | 1330(3.4) |
| Mild impairment | 6328(15.3) | 217(10.4) | 6111(15.5) |
| Moderate impairment | 8715(21.0) | 331(15.8) | 8384(21.3) |
| High impairment | 24802(59.8) | 1496(71.5) | 23306(59.2) |
| **Complex Health Care Rating**^§^ |  |  |  |
| A (best) | 9781(23.6) | 511(24.4) | 9270(23.5) |
| B | 10471(25.3) | 505(24.1) | 9966(25.3) |
| C | 11554(27.9) | 608(29.0) | 10946(27.8) |
| D (worst) | 9408(22.7) | 459(21.9) | 8949(22.7) |
| **Mobility Rating**^§¶^ |  |  |  |
| A (best) | 1985(4.8) | 100(4.8) | 1885(4.8) |
| B | 3141(7.6) | 134(6.4) | 3007(7.6) |
| C | 16999(41.0) | 924(44.1) | 16075(40.8) |
| D (worst) | 19089(46.0) | 925(44.2) | 18164(46.1) |
| **Wandering Rating^§¶^** |  |  |  |
| A (best) | 26205(63.2) | 1156(55.2) | 25049(63.6) |
| B | 3962(9.6) | 182(8.7) | 3780(9.6) |
| C | 2552(6.2) | 142(6.8) | 2410(6.1) |
| D (worst) | 8495(20.5) | 603(28.8) | 7892(20.0) |
| **Verbal Behaviour Rating^§¶^** |  |  |  |
| A (best) | 6784(16.4) | 255(12.2) | 6529(16.6) |
| B | 6479(15.6) | 293(14.0) | 6186(15.7) |
| C | 6857(16.5) | 325(15.5) | 6532(16.6) |
| D (worst) | 21094(50.9) | 1210(57.8) | 19884(50.5) |
| **Physical Behaviour Rating^§¶^** |  |  |  |
| A (best) | 11391(27.5) | 413(19.7) | 10978(27.9) |
| B | 6485(15.6) | 327(15.6) | 6158(15.6) |
| C | 5916(14.3) | 302(14.4) | 5614(14.3) |
| D (worst) | 17422(42.0) | 1041(49.7) | 16381(41.6) |
| **Number of Unplanned Hospitalisations^#^** |  |  |  |
| None | 12821(30.9) | 530(25.3) | 12291(31.2) |
| 1 | 13997(33.8) | 688(32.9) | 13309(33.8) |
| 2-4 | 12957(31.2) | 742(35.5) | 12215(31.0) |
| ≥5 | 1694(4.1) | 133(6.4) | 1561(4.0) |
| **Number of ED Presentations^#^** |  |  |  |
| 0 | 13479(32.5) | 515(24.6) | 12964(32.9) |
| 1 | 12241(29.5) | 611(29.2) | 11630(29.5) |
| 2-4 | 13048(31.5) | 752(35.9) | 12296(31.2) |
| ≥5 | 2701(6.5) | 215(10.3) | 2486(6.3) |
| **Number of GP Attendances^#^** |  |  |  |
| 0 | 1499(3.6) | 62(3.0) | 1437(3.6) |
| 1-5 | 10917(26.3) | 456(21.8) | 10461(26.6) |
| 6-15 | 19937(48.1) | 1053(50.3) | 18884(48.0) |
| ≥16 | 9116(22.0) | 522(24.9) | 8594(21.8) |
| **Number of Geriatric Medicine Physician Attendances^#^** |  |  |  |
| 0 | 36881(88.9) | 1823(87.1) | 35058(89.0) |
| 1 | 3016(7.3) | 185(8.8) | 2831(7.2) |
| ≥2 | 1572(3.8) | 85(4.1) | 1487(3.8) |

ED=Emergency Department. GP=General Practitioner. ^#^Delirium or dementia-related hospitalisation within 365 days after LTCF entry, defined as hospitalisation or ED presentation where dementia or delirium was recorded as the principal discharge diagnosis for the encounter. ^†^Conditions were ascertained using the aged care eligibility or entry into care assessments. ^‡^Medications at the chemical subgroup level (ATC 4th level, i.e. 5 digits). ^¶^A is least dependant and D is most dependant. ^§^Care needs of LTCF residents from entry into LTCF assessments. ^#^Ascertained in the 365 days prior to LTCF entry. Missing data N(%): Facility Remoteness: 80(0.2); SEIFA Index of Economic Resources: 388(0.9).

# Comparison of model performance in the training and testing samples using Harrell’s C-index

To assess for potential overfitting, model performance was compared using the Harrell’s C-index in both the training and test sets. In the training sample, the Harrell’s C-index was 0.678 (95% CI 0.672-0.684), and in the test sample, the C-index was 0.670 (95% CI 0.658-0.682), indicating good model stability with minimal overfitting.

# Supplemental Table 9. Model Performance Metrics Across Different Risk Thresholds in the Testing Sample.

| **Threshold** | **Sensitivity** | **Specificity** | **Positive Predictive Value (PPV)** | **Negative Predictive Value (NPV)** |
| --- | --- | --- | --- | --- |
| 0.025 | 96.9% | 8.9% | 5.4% | 98.2% |
| 0.05 | 73.6% | 46.7% | 6.9% | 97.0% |
| 0.10 | 30.7% | 86.8% | 11.0% | 95.9% |
| 0.15 | 12.2% | 96.5% | 15.7% | 95.3% |
| 0.20 | 4.1% | 99.0% | 18.5% | 95.0% |
| 0.25 | 1.7% | 99.7% | 20.7% | 95.0% |
| 0.35 | 0.2% | 99.9% | 19.0% | 94.9% |

**
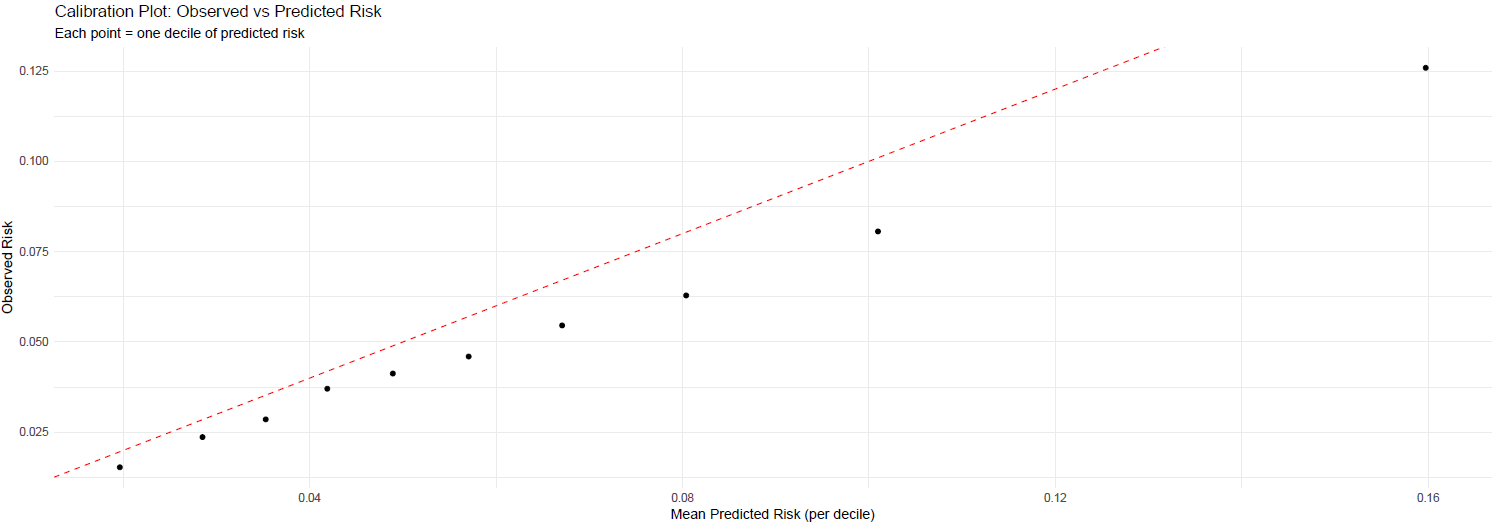
**

# Supplemental Figure 1. Calibration Plot: Observed Vs. Mean Predicted Risk (per Decile) of Delirium or Dementia-Related Hospitalisation Using the Training Sample.

**
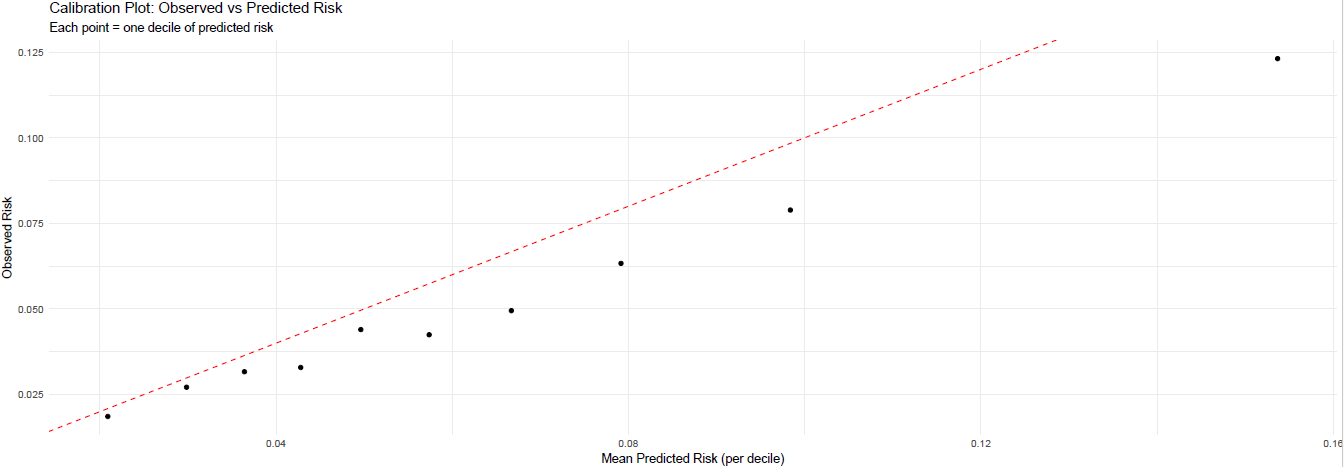
**

# Supplemental Figure 2. Calibration Plot: Observed Vs. Mean Predicted Risk (per Decile) of Delirium or Dementia-Related Hospitalisation Using Testing Sample Data.

**A
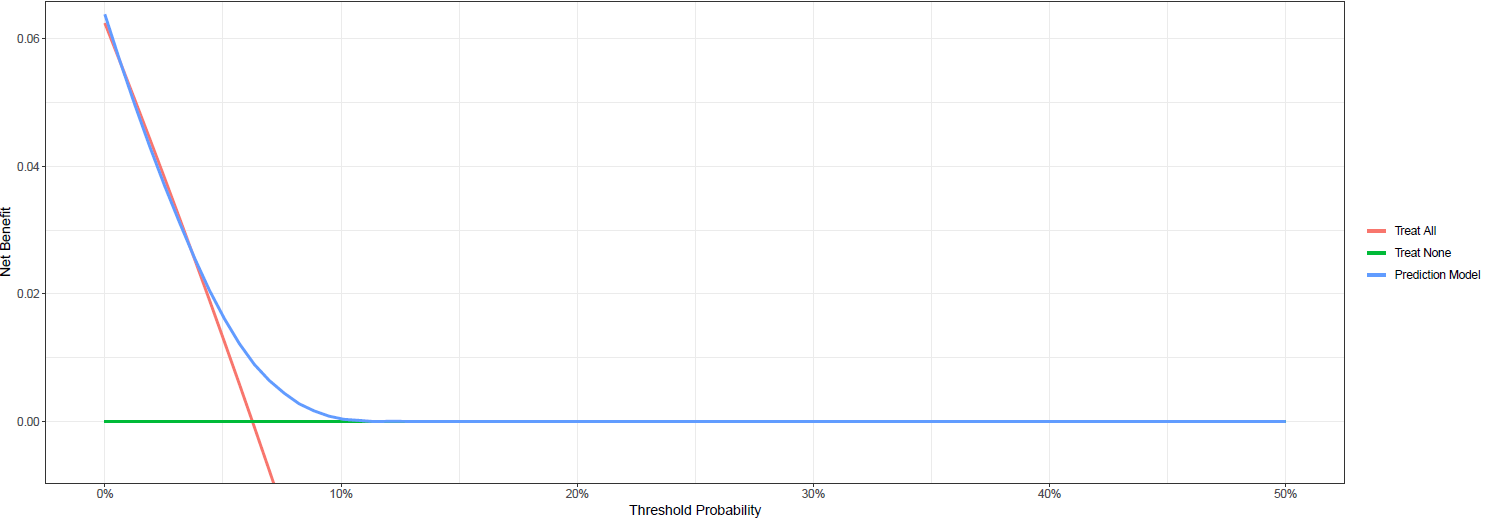
**

**B**

**
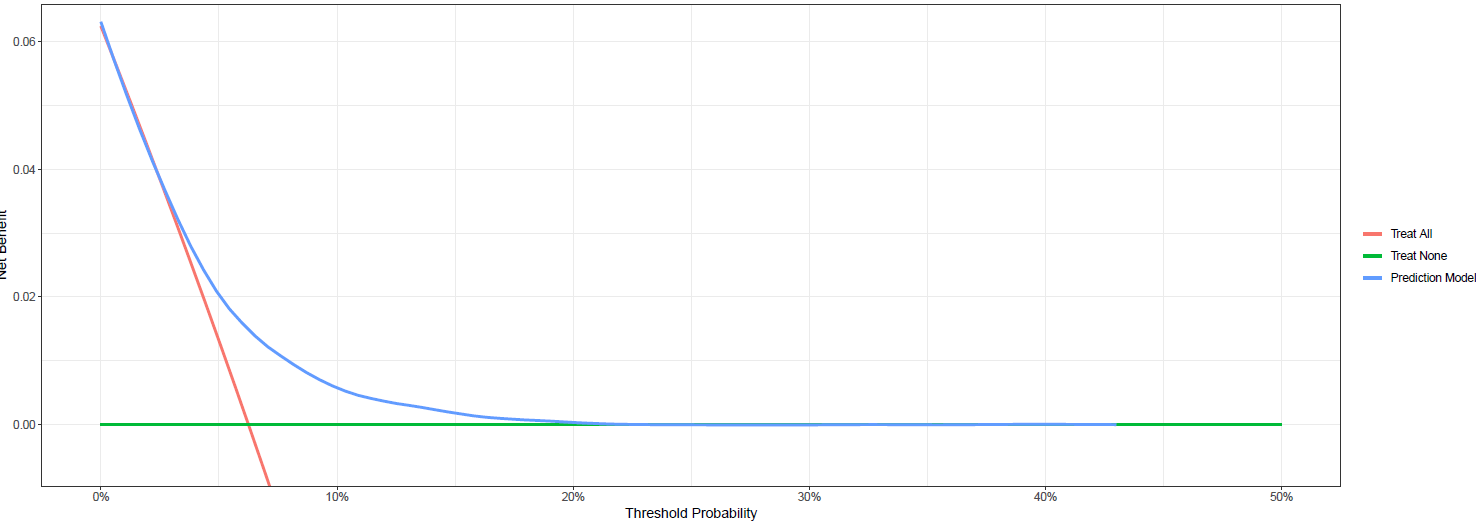
**

# Supplemental Figure 3. Clinical Decision Curve Analysis (DCA) Using the Testing Sample: (A) base model based on age and sex; (B) final model.


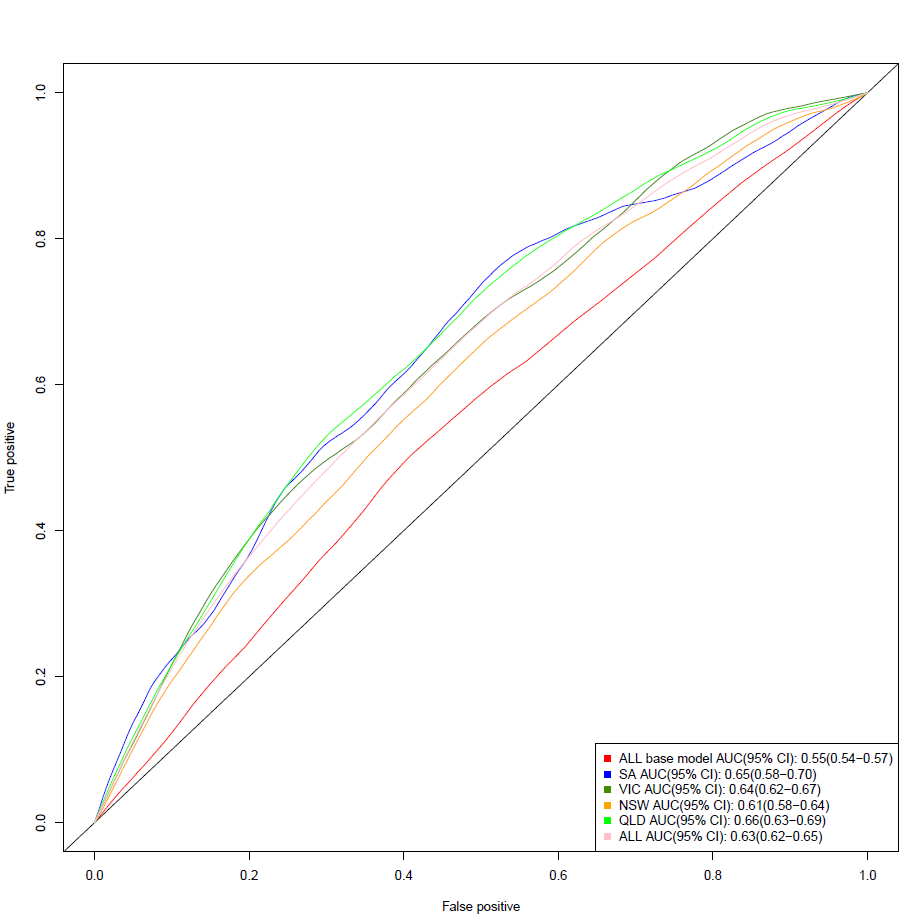


# Supplemental Figure 4. The Predictive Performance of Delirium-Related Hospitalisation Only Risk Prediction Model (Out of Sample). AUC: Area Under the Receiver Operating Characteristics Curve; CI: Confidence Interval; SA: South Australia; VIC: Victoria; NSW: New South Wales; QLD: Queensland.

# TRIPOD+AI Checklist: Prediction Model Development and Validation.


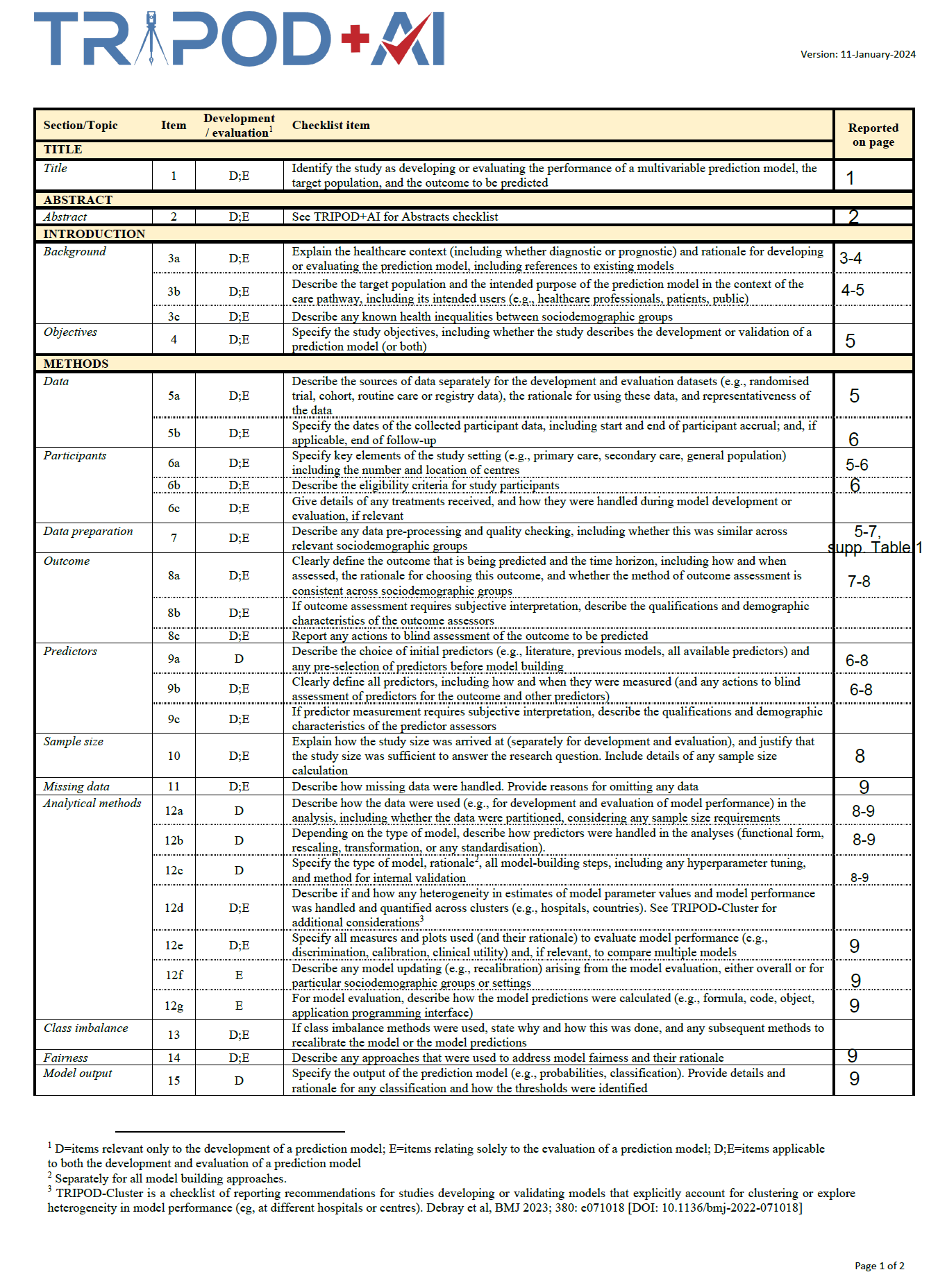


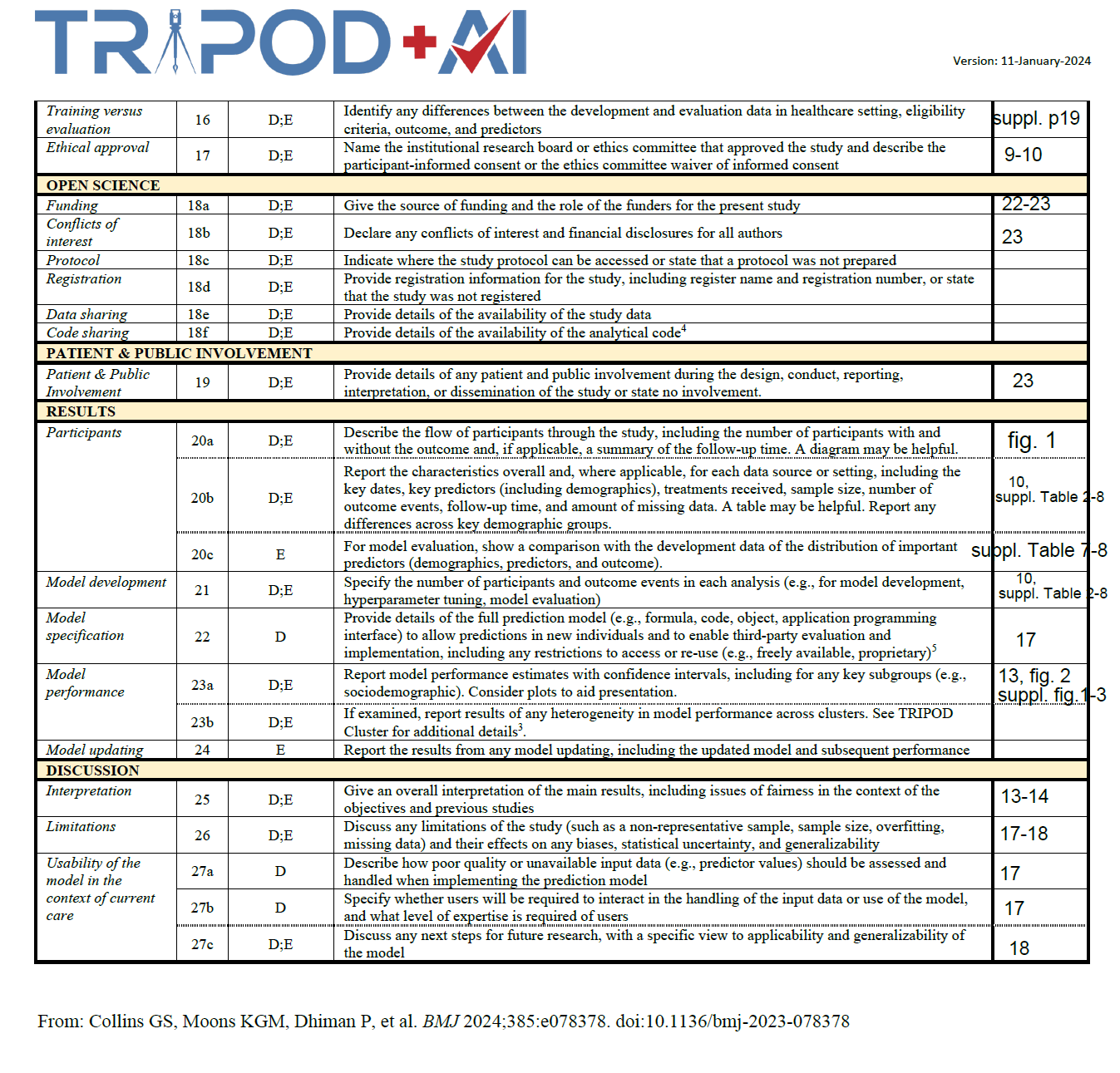


**References**

1. Moldovan M, Taylor J. Elastic Net (Model Selection). In: *Wiley StatsRef: Statistics Reference Online.*1-5.

2. Fridgeirsson EA, Williams R, Rijnbeek P, Suchard MA, Reps JM. Comparing penalization methods for linear models on large observational health data. *Journal of the American Medical Informatics Association.* 2024;31(7):1514-1521.

3. Zou H, Hastie T. Regularization and Variable Selection Via the Elastic Net. *Journal of the Royal Statistical Society Series B: Statistical Methodology.* 2005;67(2):301-320.

4. Ensor J. pmsampsize: Sample Size for Development of a Prediction Model. <https://cran.r-project.org/web/packages/pmsampsize/index.html>. Published 2023. Accessed 5 May, 2025.
